# An activator of voltage-gated K^+^ channels Kv1.1 as a therapeutic candidate for episodic ataxia type 1

**DOI:** 10.1073/pnas.2207978120

**Published:** 2023-07-24

**Authors:** Ilenio Servettini, Giuseppe Talani, Alfredo Megaro, Maria Dolores Setzu, Francesca Biggio, Michelle Briffa, Luca Guglielmi, Nicoletta Savalli, Francesca Binda, Francis Delicata, Gilles Bru–Mercier, Neville Vassallo, Vittorio Maglione, Ruben J. Cauchi, Alba Di Pardo, Maria Collu, Paola Imbrici, Luigi Catacuzzeno, Maria Cristina D’Adamo, Riccardo Olcese, Mauro Pessia

**Affiliations:** ^a^Section of Physiology, Department of Medicine, University of Perugia, Perugia 06123, Italy; ^b^Institute of Neuroscience, National Research Council, Monserrato 09042, Italy; ^c^Department of Biomedical Sciences, University of Cagliari, Monserrato 09042, Italy; ^d^Department of Life and Environmental Sciences, University of Cagliari, Monserrato 09042, Italy; ^e^Department of Physiology and Biochemistry, Faculty of Medicine and Surgery, University of Malta, Msida MSD2080, Malta; ^f^Medical Research Council Laboratory of Molecular Biology, Cambridge CB2 0QH, United Kingdom; ^g^Department of Anesthesiology and Perioperative Medicine, David Geffen School of Medicine, University of California, Los Angeles, CA 90095; ^h^Department of Fundamental Neurosciences, University of Lausanne, Lausanne 1011, Switzerland; ^i^Centre National de la Recherche Scientifique, Institut des Neurosciences Cellulaires et Intégratives, Université de Strasbourg, Strasbourg F-67000, France; ^j^College of Pharmacy, Rady Faculty of Health Sciences, University of Manitoba, Winnipeg, MB R3E 0T5, Canada; ^k^Department of Physiology, College of Medicine and Health Sciences, United Arab Emirates University, Al Ain 17666, United Arab Emirates; ^l^Istituto di Ricovero e Cura a Carattere Scientifico Neuromed, Pozzilli 86077, Italy; ^m^Department of Pharmacy–Drug Sciences, University of Bari ‘‘Aldo Moro”, 70125 Bari, Italy; ^n^Department of Chemistry, Biology and Biotechnology, University of Perugia, Perugia 06123, Italy; ^o^Department of Medicine and Surgery, Libera Università Mediterranea ‘‘Giuseppe DEGENNARO”, Casamassima 70010, Italy; ^p^Department of Physiology, David Geffen School of Medicine, University of California, Los Angeles, CA 90095

**Keywords:** Kv1.1(*KCNA1*), Kv1.2(*KCNA2*), niflumic acid, episodic ataxia type 1, OMIM160120

## Abstract

Loss-of-function mutations in the *KCNA1*(Kv1.1) gene cause episodic ataxia type 1 (EA1), a neurological disease characterized by cerebellar dysfunction, ataxic attacks, persistent myokymia with painful cramps in skeletal muscles, and epilepsy. Precision medicine for EA1 treatment is currently unfeasible, as no drug that can enhance the activity of Kv1.1-containing channels and offset the functional defects caused by *KCNA1* mutations has been clinically approved. Here, we uncovered that niflumic acid (NFA), a currently prescribed analgesic and anti-inflammatory drug with an excellent safety profile in the clinic, potentiates the activity of Kv1.1 channels. NFA increased Kv1.1 current amplitudes by enhancing the channel open probability, causing a hyperpolarizing shift in the voltage dependence of both channel opening and gating charge movement, slowing the OFF-gating current decay. NFA exerted similar actions on both homomeric Kv1.2 and heteromeric Kv1.1/Kv1.2 channels, which are formed in most brain structures. We show that through its potentiating action, NFA mitigated the EA1 mutation–induced functional defects in Kv1.1 and restored cerebellar synaptic transmission, Purkinje cell availability, and precision of firing. In addition, NFA ameliorated the motor performance of a *knock-in* mouse model of EA1 and restored the neuromuscular transmission and climbing ability in *Shaker* (Kv1.1) mutant *Drosophila melanogaster* flies (*Sh^5^*). By virtue of its multiple actions, NFA has strong potential as an efficacious single-molecule-based therapeutic agent for EA1 and serves as a valuable model for drug discovery.

## Significance

The Kv1.1 channels control critical neuronal functions. Indeed, Kv1.1 dysfunction results in EA1 [OMIM #160120], a disorder characterized by cerebellar ataxia and epilepsy. Ideally, a treatment for EA1 should restore Kv1.1 activity, ameliorating neuronal excitability, synaptic and cerebellar function, and motor performance. Unfortunately, no such drugs exist. Here, we show that niflumic acid (NFA), a commercially available analgesic and anti-inflammatory drug, enhances the activity of not only Kv1.1 but also the Kv1.2 channel, which usually assembles with Kv1.1. We offer insights into the biophysical mechanisms of NFA action with neurophysiological and behavioral evidence showing that NFA ameliorates EA1-associated dysfunctions in Kv1.1 channel gating, synaptic transmission, neuronal excitability, and motor deficits. We believe NFA holds therapeutic value for patients with *Kv1.1 channelopathy*.

Potassium channels constitute one of the largest groups of signaling molecules encoded by the human genome (1, 2). A noteworthy example is Kv1.1 (3), a voltage-gated, K^+^-selective channel that is widely expressed throughout the nervous system. Kv1.1 subunits can assemble with Kv1.2 to form heteromeric channels (4, 5). They regulate several crucial functions including neuronal excitability, action potential threshold, waveforms, frequency, and neurotransmitter release at axon terminals. Their critical pathophysiological relevance is highlighted by the overt phenotype displayed by either transgenic animals or patients carrying mutations in pertinent genes, which are often associated with severe diseases in humans (6).

Episodic ataxia type 1 (EA1) was the first discovered *K_V_ channelopathy* and is caused by missense mutations in the *KCNA1* (Kv1.1) gene (7, 8). Interictal myokymia and generalized ataxia attacks induced by emotions or stress are key features of the illness. However, since its first description, its phenotypic spectrum has widened considerably. Severe atypical symptoms, including long-lasting attacks of ataxia, painful cramps, recurrent status epilepticus, malignant hyperthermia, insomnia, skeletal deformities, and cognitive impairment, make EA1 a severe illness in some patients (9). Heterologous expression studies have shown that heterozygous EA1 mutations result in haploinsufficiency, often associated with reduced voltage sensitivity and open probability of Kv1.1 channels (9). Notably, a knock-in mouse strain harboring the heterozygous V408A mutation (*Kv1.1^V408A/+^*), responsible for EA1 in patients, recapitulates the main features of the human disease and represents a valuable animal model (10, 11). These animals display abnormal cerebellar basket cell–Purkinje cell (BC–PC) synaptic transmission associated with prolonged action potential duration at BC presynaptic terminals, increased GABA release from BC terminals, decreased spontaneous PC firing, and impaired motor performance (10, 12). This evidence confirmed the adequacy of the disease model originally proposed by our research group (c.f. figure 7 in ref. 13).

Currently, EA1 patients are treated with acetazolamide (ACTZ); however, its efficacy decreases over time (14), and several patients exhibit resistance or develop adverse side effects such as nephrolithiasis, hyperhidrosis, paresthesia, rash, diffuse weakness, and gastrointestinal discomfort, which result in therapy discontinuation (14). Moreover, ACTZ treatment is contraindicated in individuals with liver, renal, or adrenal insufficiency (14–16). Precision medicine for EA1 should restore proper Kv1.1 activity, thereby ameliorating neuronal excitability, synaptic functionality, cerebellar function, and motor performance. Identifying repurposable drug candidates capable of activating Kv1.1 and Kv1.2 channels has proven to be challenging.

Here, we found that niflumic acid (NFA) acts as a powerful Kv1.1 activator, enhancing the voltage sensitivity, open probability of the channel and improving macroscopic current kinetics. NFA significantly counteracted EA1 mutation–induced Kv1.1 channel dysfunction and GABA*ergic* tone in the cerebellum of *Kv1.1^V408A/+^* mice. In addition, NFA ameliorated the motor performance of this well-established mouse model of EA1, as well as the neuromuscular transmission, and behavioral signs of *Drosophila melanogaster* with a similar missense mutation in their *Shaker* K^+^ channel gene (17, 18). These findings suggest that NFA may be a therapeutic opportunity for EA1 and an excellent prototype for designing compounds with improved potency, pharmacokinetics, and reduced off-target effects.

## Results

### NFA Increases the Open Probability of Kv1.1 Channels and Modulates Macroscopic Current Kinetics.

**Fig. 1.**
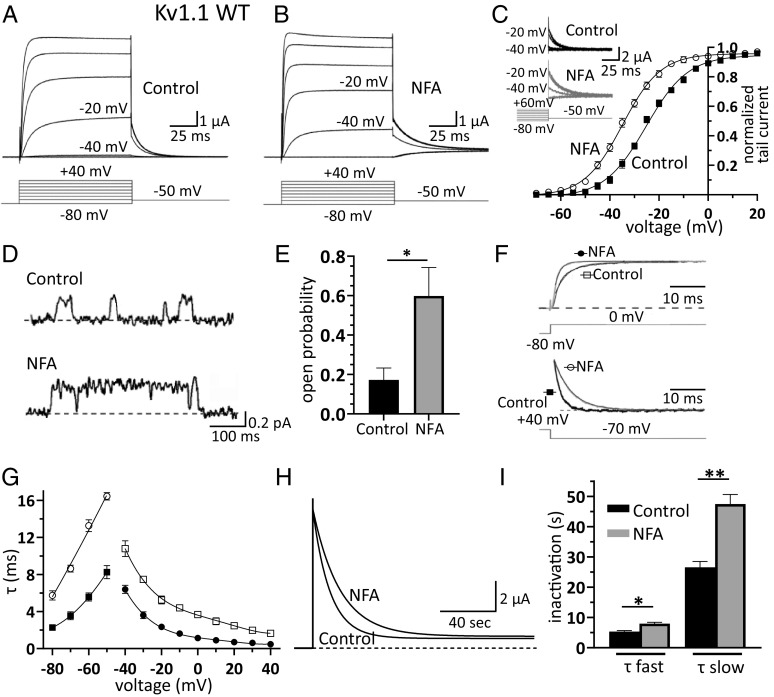
NFA is a Kv1.1 channel opener. (*A* and *B*) Families of K^+^ current traces recorded from oocytes expressing Kv1.1 under control conditions (*A*) and after bath application of 100 µM NFA (*B*). In the TEVC mode, membrane potential was depolarized from −80 mV to 40 mV (ΔV = 20 mV) from a holding potential of −80 mV (*Inset*). The current recordings at −40 and −20 mV are indicated for direct comparison of the control vs. NFA. (*C*) Plot of normalized peak tail current amplitudes recorded at −50 mV as a function of depolarizing prepulse potentials (ΔV = 5 mV) and constructed before (*closed squares*) and after (*open circles*) NFA superfusion. Data points were fitted with a Boltzmann function from which the half-maximal activation voltage (*V*_1/2_) and slope factor (*k*) were calculated. Note that NFA shifted I_tail_/V relationships toward more hyperpolarized potentials (mean ± SEM, n = 7). The *Insets* show families of Kv1.1 tail currents recorded according to the voltage protocol depicted below under control conditions and with 100 µM NFA [the best fit parameters are control (squares): V_1/2_ = −24.9 mV and k = 8.1; NFA (circles): V_1/2_ = −34.7** mV and k = 7.6]. (*D*) Single-channel currents recorded in cell-attached configuration from Kv1.1-expressing oocytes in the absence (*control*) and presence of 100 µM NFA in the pipette solution (*dashed lines indicate the channel closed state*). Channel openings were evoked by depolarizing pulses at −30 mV lasting 500 ms and delivered every 5 s (holding potential: −80 mV; sample traces were filtered at 0.2 kHz). (*E*) Mean single-channel open probability assessed at −30 mV in the absence (*control*) and presence of NFA. Single-channel open probability was calculated from the closed and open state areas of the current amplitude histograms, estimated by performing a double Gaussian fit. Data are mean ± SEM; n = 6; *P < 0.05. (*F*) Sample Kv1.1 current traces recorded at 0 mV (*upper traces*) or during a repolarizing step to −70 mV (*lower traces*) under control conditions (*black traces*) or in the presence of 100 µM NFA (*gray traces*). Currents in the presence and absence of NFA were normalized to their maximum value to directly compare changes in kinetics. Continuous lines superimposed to traces represent best fits with monoexponential functions. (*G*) Plot of activation (*open squares: control; closed circles: NFA*) and deactivation (*closed squares: control; open circles: NFA*) time constants resulting from the best fit of current traces with monoexponential functions, calculated under control conditions and with 100 µM NFA. (*H*) Superimposed Kv1.1 currents showing the time course of slow inactivation induced by a 210-s long depolarizing step to 0 mV, under control conditions and during bath application of 100 µM NFA. The continuous black lines superimposed to current decays show the best fit with double-exponential functions from which the fast and slow time constants of inactivation were calculated, averaged and plotted as bar graphs (*I*); data are mean ± SEM (n = 8; *P < 0.05 **P < 0.001, paired *t* test).

*Xenopus* oocytes are a unique model system that faithfully and efficiently translate foreign genetic information and have been largely used to express membrane proteins for structure–function analyses of K^+^ channels. To study the effect of NFA on Kv1.1 function, we expressed human Kv1.1 in oocytes and performed electrophysiological recordings in the two-electrode voltage-clamp (TEVC) mode. Kv1.1 current amplitudes were enhanced by extracellular NFA superfusion (Fig. 1 *A* and *B*). This effect typically required 2 to 5 min to reach a steady-state level and was reversible upon washing. The effect of NFA on K^+^ currents was particularly evident at negative potentials, between −50 mV and −20 mV (*SI Appendix*, Fig. S1 *A* and *B*), where outward currents increased several folds in a concentration–dependent manner (*SI Appendix*, Figs. S1*B* and S2 *A* and *B*). In contrast, at depolarized potentials greater than 0 mV, the effect of NFA on the current amplitudes was negligible (*SI Appendix*, Fig. S1 *A* and *B*). The steady-state current–voltage relationship, constructed before and after drug application, showed that NFA facilitated Kv1.1 activation (Fig. 1*C*) by shifting the half-activation voltage (V_1/2_) toward more hyperpolarized potentials (*SI Appendix*, Table S1) in a dose-dependent manner (*SI Appendix*, Fig. S2 *A* and *B*).

To assess its possible effect on the oocytes’ endogenous channels, NFA was applied to cells injected with distilled H_2_O. NFA had no or negligible effects on the endogenous currents (*SI Appendix*, Fig. S3), compared with those exerted on Kv1.1 currents, indicating that NFA does not modulate the gating of Kv1.1 channels by acting on Ca^2+^-activated chloride channels 16/anoctamin-1 (ANO1; TMEM16A), which are endogenously expressed by oocytes. To validate the oocyte data in another expression system, we performed whole-cell patch-clamp recordings from HEK293 cells transfected with Kv1.1 channels before and after NFA application. Consistent with the findings in oocytes, we recorded a significant NFA-mediated increase in Kv1.1 currents at negative potentials, associated with a shift in the voltage dependence toward more hyperpolarized potentials, and slower deactivation kinetics (*SI Appendix*, Fig. S4 *A–D*). To further validate our findings using a neuronal cell system, we tested the effects of NFA using Neuro-2a cells (N2a). This cell type exhibits neuronal stem cell properties (19) and can differentiate into neurons able to discharge action potentials (*SI Appendix*, Fig. S5 *A* and *B*). Notably, these neurons endogenously express Kv1.1 channels at high levels, as well as other voltage-gated K^+^ channel types (19). Patch-clamp recordings showed that NFA increased the amplitude of the K^+^ current at −10 mV by approximately twofold, negatively shifted the voltage dependence of activation by approximately 15 mV, and slowed deactivation kinetics (*SI Appendix*, Fig. S5 *C–I*).

The NFA-induced negative shift of steady-state voltage activation obtained from macroscopic tail currents (Fig. 1*C*) suggests that the drug likely affects the Kv1.1 single-channel open probability. Thus, cell-attached patch-clamp recordings were performed using oocytes expressing Kv1.1, in the absence and presence of NFA in the pipette solution (Fig. 1*D*). Under both conditions, amplitude histograms were constructed from the current traces originating from patches containing only one active channel. The channel open probability and unitary current were calculated from the areas and mean currents resulting from the fitting of the histograms with double Gaussian functions (*SI Appendix*, Fig. S6 *A* and *B*). NFA increased the open probability of Kv1.1 channels by approximately threefold at −30 mV (Fig. 1*E*). The Kv1.1 unitary currents were then plotted as a function of membrane potential, and channel conductance was estimated by linear fits, which showed that NFA did not affect the slope conductance of Kv1.1 channels (*SI Appendix*, Fig. S6*C*). Unlike the significant effect observed with NFA added to the pipette solution, we could not detect any changes in channel opening probability when a similar concentration of NFA was added to the extracellular bath solution while recording the openings in the cell-attached configuration (channel confined within the pipette), or when NFA was applied to the intracellular side of the channel in the inside-out patch configuration. Taken together, these results suggest that the binding site for NFA is likely on the extracellular side of the channel. The plot of time constants of ionic current activation and deactivation, as a function of membrane potential, showed that NFA slowed Kv1.1 channel deactivation, instead accelerating channel activation (Fig. 1 *F* and *G*). Next, we investigated the effects of NFA on slow inactivation since homomeric Kv1.1 channels are characterized by a slow inactivation process that affects the firing rate and shape of action potentials during prolonged neuronal activity (20). Thus, possible modifications of this distinct channel property by NFA may be mechanistically and therapeutically relevant. To assess the action of NFA on Kv1.1 slow inactivation, the cell membrane potential was held at −80 mV, and currents were evoked by depolarization at +40 mV for 3.5 min. The superimposed current traces clearly showed that Kv1.1 channels underwent a slower inactivation process in the presence of NFA (Fig. 1*H*). To quantify this effect, the decay phase of the currents was fitted using double exponential functions. This analysis showed that NFA increased the fast and slow time constants by approximately 50% and 80%, respectively (Fig. 1*I* and *SI Appendix*, Table S1). These results indicate that NFA slows down the entry of Kv1.1 channels to the inactivated state.

We next tested whether NFA could exert similar effects on homomeric Kv1.2 channels, a mammalian homologue of Kv1.1 (21, 22). NFA shifted the voltage dependence of steady-state activation leftward, accelerated activation and slowed down the deactivation and inactivation kinetics of Kv1.2 channels expressed in oocytes (*SI Appendix*, Table S1). Patch-clamp recordings of HEK293 cells transfected with Kv1.2 showed that NFA enhanced K^+^ currents at negative potentials, although at depolarized potentials, the effect of NFA on the current amplitudes was negligible (*SI Appendix*, Fig. S7). However, Kv1.2 channels expressed in mammalian cells exhibit two distinct gating modes (‘‘slow’’ and ‘‘fast’’) (23, 24), a property that is lost when the channel is expressed in oocytes. NFA significantly shifted the voltage dependence of steady-state activation leftward and slowed down the deactivation of Kv1.2 channels expressed in HEK293 cells (*SI Appendix*, Fig. S7). These effects of NFA were observed on Kv1.2 channels exhibiting either the *slow* (*SI Appendix*, Fig. S7 *A* and *D*) or *fast*-*gating mode* (*SI Appendix*, Fig. S7 *E–H*). Notably, Kv1.2 commonly forms heteromeric channels with Kv1.1 in the nervous system (21, 22) and confers the dual gating mode to heteromeric channels comprising Kv1.1 and Kv1.2 subunits. NFA exerted similar actions also on Kv1.1/Kv1.2 channels in either the *slow* (*SI Appendix*, Fig. S8 *A–D*) or *fast*-*gating mode* (*SI Appendix*, Fig. S8 *E–H*). To establish whether other *fenamates* structurally related to NFA were also able to modulate Kv1.1-mediated currents, we assessed the effect of *mefenamic acid* (MFA). MFA at a relatively high concentration (300 µM) led to a modest (~2 mV) shift in the steady-state voltage activation of the current (V_1/2_ control = −28.7 ± 1.3 mV; V_1/2_ MFA = −30.8 ± 1.2 mV; n = 4, *P* < 0.05) and increased the rate of current activation (the activation time constant at 0 mV was 4.10 ± 0.11 ms (control) vs. 1.76 ± 0.14 ms (MFA; n = 4; *P* < 0.05). Collectively, the aforementioned evidence indicates that NFA is a Kv1.1 and Kv1.2 activator, which enhances the channel’s voltage sensitivity.

### NFA Enhances *Shaker K^+^ Currents Amplitudes and Modulates the* Voltage Dependence and Kinetics of Gating Charges.

The *Shaker* K^+^ channel (*Sh*) is a Kv1.1 ortholog (3, 17, 18, 25, 26). To record tail currents and assess the effect of NFA on the voltage dependence of the *Shaker* channel, we expressed in oocytes a *Δ*6–46 deletion mutant (*Sh–IR*), which removes fast N-type inactivation (*IR*). NFA shifted the IV relationships of *Shaker Sh–IR* channels to more hyperpolarized potentials (*SI Appendix*, Fig. S9 *A–G*). We also tested the effects of NFA on wild-type (WT) *Shaker* currents, which were enhanced several folds at negative potentials, while its effect at positive potentials was negligible (*SI Appendix*, Fig. S10 *A–D* and *G*). Both, NFA and MONNA inhibit ANO1 channels (27). Nevertheless, the application of MONNA did not affect the *Shaker* K^+^ current amplitude (*SI Appendix*, Fig. S10 *E–G*), supporting the notion that NFA does not modulate the gating of *Shaker* channels by acting on ANO1 channels.

The effect of NFA on the voltage-dependent activation of both *Shaker* and human Kv1.1 channels suggested that the drug likely affects the properties of the voltage-sensing domain. To explore this possibility, we tested its effect on gating currents, which are generated by the movement of the voltage sensor in response to voltage changes. The *Shaker* channel proved to be an excellent candidate for studying gating currents because of its high level of expression in Xenopus oocytes. To avoid significant contamination on gating current measurements by both the fast N-type inactivation and ionic current, we used the *Shaker* channel *Sh–IR–WF* (28–31), which in addition to the *Δ*6–46 deletion carries the W434F mutation (*WF*) in the pore that results in nonconducting channels (hereafter named *Sh–IR–WF* channel) (32). Using the *cut-open* oocyte voltage-clamp technique (33, 34), gating currents from the *Sh–IR–WF* channel were recorded before and after NFA application (*SI Appendix*, Fig. S11*A*), and analyzed by integrating the ON gating current (I_g–ON_) and plotting the gating charge (Q) as a function of membrane potentials. Notably, NFA shifted the Q–V relationship and V_1/2_ toward more hyperpolarized membrane potentials, suggesting that the drug affects Kv1.1 function by modulating the voltage sensor activation (*SI Appendix*, Fig. S11 *B* and *C*). Next, we evaluated the kinetics of gating charge transitions toward both the activated and resting positions, by estimating the decay rates of I_g–ON_ and I_g–OFF_, respectively. The normalized and superimposed I_g–ON_ and I_g–OFF_ showed that NFA slowed the decay rate of I_g–OFF_, whereas the kinetics of I_g–ON_ were not affected (*SI Appendix*, Fig. S11*D*). These effects were quantified by fitting both the I_g–ON_ and I_g–OFF_ decay with a single exponential function and plotting the mean time constants as a function of the membrane potential. This analysis confirmed that the I_g–ON_ decay time course was largely unaffected by NFA (*SI Appendix*, Fig. S11*E*, *Upper*), whereas the I_g–OFF_ decay was slowed throughout the voltage range examined (*SI Appendix*, Fig. S11*E*, *Lower*). Overall, the left-shifted Q–V and slower I_g–OFF_ kinetics are consistent with the hypothesis that NFA stabilizes the voltage sensors in their activated state.

### NFA Mitigates the Functional Defects Induced by the EA1 Mutation V408A.

**Fig. 2.**
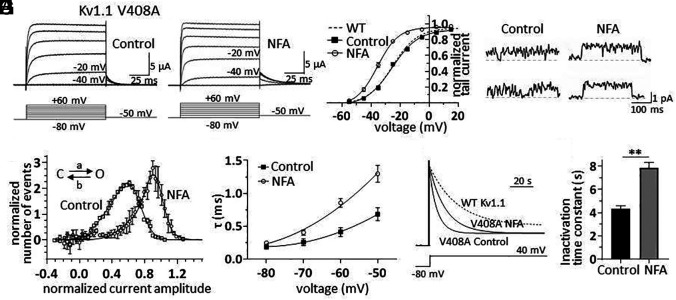
NFA counteracts Kv1.1 channel dysfunction induced by the EA1 mutation V408A. (*A* and *B*) Families of K^+^ currents recorded from oocytes expressing Kv1.1-V408A under control conditions (*A*) and during bath application of 100 µM NFA (*B*); currents were elicited by applying the voltage protocol shown below each sample trace. The current traces recorded at −40 mV and −20 mV are labeled for direct comparison of the effect of NFA. (*C*) Plot of normalized mean tail current amplitudes as a function of membrane potential obtained from Kv1.1-V408A-expressing oocytes (n = 6) by applying depolarizing voltage steps from −80 mV to +60 mV (ΔV = 10 mV) followed by a repolarization to −50 mV. The solid lines represent fits of the experimental data points with a Boltzmann relationship. The best fit parameters are V_1/2_ = −24.8 mV and k = 8.2 mV under control conditions and V_1/2_ = −35.0** mV and k = 6.6 mV in the presence of NFA. The dashed line represents the steady-state activation of WT Kv1.1 channels taken from Fig.1*C*. (*D*) Single-channel recordings in cell-attached configuration from oocytes expressing Kv1.1–V408A in the absence (*Left*) or presence (*Right*) of 100 µM NFA in the pipette solution. Due to the “*flickery*” behavior of the channel, depolarizing pulses at 20 mV were used to better record the openings. The voltage steps lasted for 500 ms and were delivered every 5 s from a holding potential of –80 mV. The traces were filtered at 0.2 kHz. (*E*) Mean *point*-*by*-*point* open channel current amplitude histograms constructed from single-channel recordings obtained at 20 mV in the absence (*open squares*) and presence (*open circles*) of 100 µM NFA. The histograms were fitted with a beta distribution describing a fast-flickering process due to the passage of the channel from a closed to an unstable open state. For fitting purposes, the beta distribution was convolved with a Gaussian function having mean zero and SD assessed from the closed state amplitude histogram. The best fit of forward and backward rate constants were control conditions: a = 2,831 s^−1^ and b = 1,886 s^−1^; NFA: a = 1,009 s^−1^ and b = 131 s^−1^. (*F*) Plot of deactivation time constants as a function of membrane potential calculated in the absence (*black squares*) and presence (*open circles*) of 100 µM NFA. (*G*) Normalized and superimposed current traces recorded from oocytes expressing Kv1.1–V408A channels in response to 100-s long depolarizing step to 0 mV, under control conditions and after the application of 100 µM NFA. The dashed trace, showing WT Kv1.1 current, was superimposed for direct comparison of slow inactivation. (*H*) Bar graph of mean time constants of slow inactivation calculated from monoexponential fits of current decay in the absence (*black bar*) and presence of NFA 100 µM (*gray bar*). Data are mean ± SEM (n = 6 to 8; *P < 0.05 **P < 0.001, paired *t* test).

Given the significant effect of NFA in enhancing Kv1.1 currents, we aimed to establish whether this drug could amend the altered Kv1.1 delayed-rectifier function underlying EA1 (6, 9). We focused our investigation on Kv1.1 channels carrying the V408A mutation (Kv1.1–V408A) since *i*) it was identified in patients with EA1 (7); *ii*) alters channel gating (8, 13, 35), and *iii*) a pertinent rodent model of EA1 has been generated by introducing a V408A mutation in the mouse genome (*Kv1.1^V408A/+^*) (10, 11), which enabled subsequent ex vivo and in vitro studies (*see below*). Therefore, similar to the WT Kv1.1 channels, we tested the effects of NFA on Kv1.1–V408A current amplitudes, voltage dependence, single-channel properties, deactivation, and inactivation kinetics. Applying NFA to oocytes expressing the mutant channels enhanced Kv1.1–V408A current amplitudes (Fig. 2 *A* and *B*), and shifted the steady-state activation to more hyperpolarized potentials of both homomeric (Kv1.1; Fig. 2*C*) and heteromeric channels containing Kv1.1–V408A subunits (*SI Appendix*, Table S1). At the single-channel level, Kv1.1–V408A currents showed closing events such that the full open level was hardly observable as compared to WT channels (Fig. 2*D*) (13). NFA ameliorated the fast-flickering behavior characteristic of mutant channels (13), by conferring a single-channel feature much more like that observed in WT channels (Fig. 2*D*). To quantify this effect thoroughly, we constructed the open-channel current amplitude distributions from Kv1.1–V408A single-channel recordings with and without NFA and interpreted these distributions in terms of fast transitions of the channel between the open and closed states by fitting them with a *beta function* (solid lines in Fig. 2*E*) (36–38). The fit indicated that NFA slowed by approximately 14-fold (from 1,886 s^−1^ to 131 s^−1^) the rate constant from the open to closed state (*b* in the scheme of Fig. 2*E*). Thus, NFA strongly disfavors the entry to the closed state, which is instead promoted by the V408A mutation. Kv1.1–V408A channels are also characterized by faster deactivation and inactivation kinetics than the WT (13, 35). Similar to what was found for WT channels, NFA slowed the rates of current deactivation (Fig. 2*F*) and inactivation of both homomeric (Fig. 2 *G* and *H*) and heteromeric channel types (*SI Appendix*, Table S1).

### NFA Inhibits Both Evoked and Spontaneous IPSCs (sIPSCs) Recorded from Cerebellar Purkinje Cells.

**Fig. 3.**
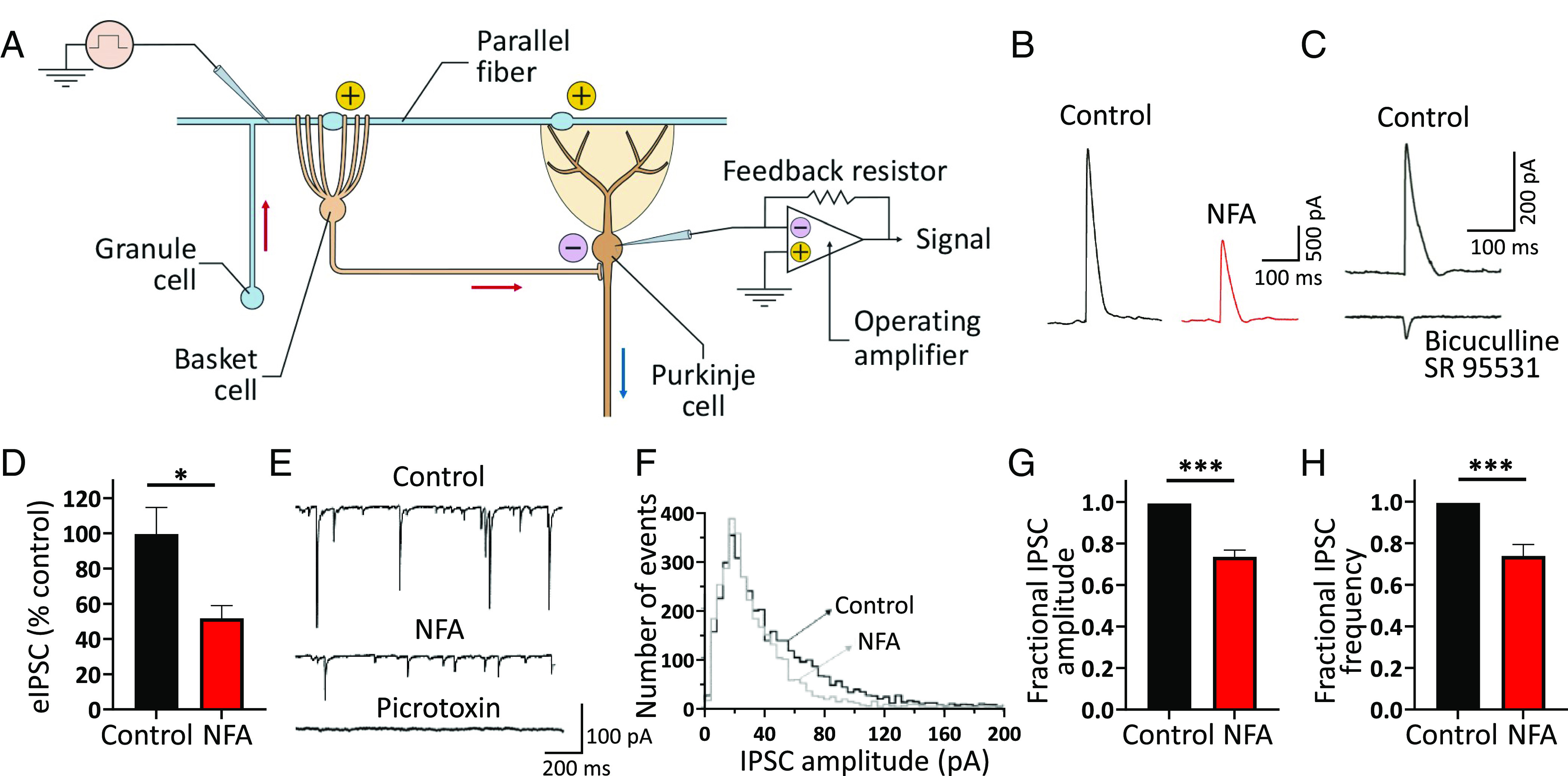
NFA curtails GABA*ergic* tone on cerebellar Purkinje neurons. (*A*) Schematic diagram showing a cerebellar granule cell (GC, *pale blue*), and parallel fiber (PF, *pale blue*) making excitatory (+) synaptic contact with the dendritic tree of both a basket cell (BC, *pale brown*) and Purkinje cell (PC*, brown*). The BC’s axon creates a GABA*ergic* inhibitory (−) synapse in the PC axon initial segment area. IPSCs were triggered by stimulation of PF (*blue pipette on the left*-*hand side*) and recorded from a PC using the whole-cell patch-clamp configuration (*blue pipette on the right*-*hand side*). (*B*) Averaged sample traces showing parallel fiber-evoked inhibitory responses (eIPSC) and recorded from a voltage-clamped PC under control conditions and during bath application of 100 µM NFA. Note that the amplitude of eIPSC was significantly reduced by NFA. (*C*) Averaged eIPSC recorded under control conditions (*upper trace*) and during bath co-application of bicuculline (20 μM) and SR95531 (*gabazine,* 20 μM) (*lower trace*). Note that the eIPSC was abolished by superfusion with these GABA_A_ inhibitors, and only a small residual EPSC component was apparent (*lower trace*). (*D*) Bar graph showing the averaged effect of NFA on the eIPSC amplitude. Data are shown as percentage of the control (mean ± SEM; n = 5; *P < 0.01, paired *t* test). (*E*) Representative traces showing sIPSCs recorded from a PC at −70 mV of applied potential under control conditions (*top trace*) and following bath application of 100 µM NFA (*middle trace*). Note that the application of 100 µM picrotoxin, an antagonist of GABA_A_ receptors, inhibited sIPSCs (*lower trace*). (*F*) Probability density function of sIPSC amplitude build from traces recorded from the same neuron shown in panel (*E*), under control conditions (*black line*) and in the presence of 100 µM NFA (*gray line*). (*G* and *H*) Bar plots showing normalized mean fractional reduction of sIPSC amplitude (*G*) and frequency (*H*) following bath application of 100 µM NFA (mean ± SEM; n = 7; *P < 0.01, ***P < 0.0001; paired *t* test).

PCs are the sole output neurons of the cerebellar cortex; their proper activity is crucial for motor coordination. Kv1.1 channels are highly expressed at the nerve terminals of cerebellar basket cells (BCs) where they control GABA release onto PCs (5). In the cerebellar cortex, granule cell axons project into the molecular layer and form parallel fibers (PFs), which form excitatory synapses with PCs, stellate cells (SCs), and BCs (Fig. 3*A*). Inhibitory stellate and basket interneurons, in turn, create GABA*ergic* synapses on PCs by implementing a feedforward inhibitory mechanism in the cerebellar cortex (Fig. 3*A*). Thus, PFs stimulation results in a biphasic current response from PCs, consisting of a monosynaptic, faster excitatory postsynaptic current (EPSC) followed by a slower disynaptic inhibitory postsynaptic current (IPSC) (39). To assess the effect of NFA on PF-activated GABA*ergic* currents in PCs, patch-clamp recordings were performed for PCs in acute cerebellar slices and IPSCs were recorded with a Cs-based low-chloride intracellular solution in response to electrical stimulation of PFs in the molecular layer (Fig. 3*A*). PCs were voltage-clamped at the reversal potential of EPSCs (0 mV) to minimize the EPSC-mediated component of the response. Evoked IPSCs (eIPSCs) appeared as outward currents under these experimental conditions (Fig. 3*B*). Bath application of NFA reduced the amplitude of eIPSCs recorded from PCs by approximately 50% (Fig. 3 *B* and *D*). The GABA*ergic* nature of eIPSCs (Fig. 3*C*, *upper trace*) was confirmed by the coapplication of bicuculline (20 μM) and SR95531 (*gabazine,* 20 μM), two GABA_A_ receptor antagonists that completely inhibited eIPSCs, while leaving only a residual EPSC component (Fig. 3*C*, *lower trace*). These findings highlight a potential new role of NFA in modulating GABA*ergic* transmission at BC–PC synapses in the cerebellar cortex.

In contrast to WT animals, *Kv1.1^V408A/+^* mice display abnormal cerebellar BC–PC synaptic transmission, with greater amplitude and frequency of sIPSCs than WT mice when recorded from cerebellar PCs (10). However, the amplitude or frequency of miniature IPSCs and the BC firing frequency did not differ between the groups (10). These defects are likely due to a V408A mutation–induced decrease in the availability of Kv1.1 channels and abnormal delayed rectifier function at BC terminals (39). Thus, we hypothesized that NFA can activate Kv1.1-containing channels and correct V408A-induced channel dysfunction, which would translate into its ability to restore the sIPSCs to amplitudes and frequencies similar to those observed in WT PCs. *Kv1.1^V408A/+^* PCs were voltage-clamped at −70 mV, and sIPSCs were recorded using patch pipettes containing CsCl under control conditions followed by bath application of NFA (Fig. 3*E*). Under these conditions, the GABA*ergic* nature of the sIPSC was confirmed by applying the GABA_A_ receptor inhibitor picrotoxin (100 µM, n = 4; Fig. 3*E*). The plot of the event amplitude distributions showed that NFA strongly reduced the number of high-amplitude sIPSCs, while leaving the number of low-amplitude events unaltered (Fig. 3 *E* and *F*). Moreover, NFA reduced both the amplitude (~36%; Fig. 3*G*) and frequency (~25%; Fig. 3*H*) of sIPSCs recorded from *Kv1.1^V408A/+^* PCs. Overall, these results suggest that NFA reduces the synaptic release of GABA onto PCs and ameliorates BC–PC synaptic transmission in the cerebellum of mutant mice by enhancing the openings of Kv1.1 and Kv1.2 channels and reducing the influx of Ca^2+^ ions at presynaptic BC terminals.

### NFA Enhances the Precision of Firing and Number of Spontaneously Active PCs.

**Fig. 4.**
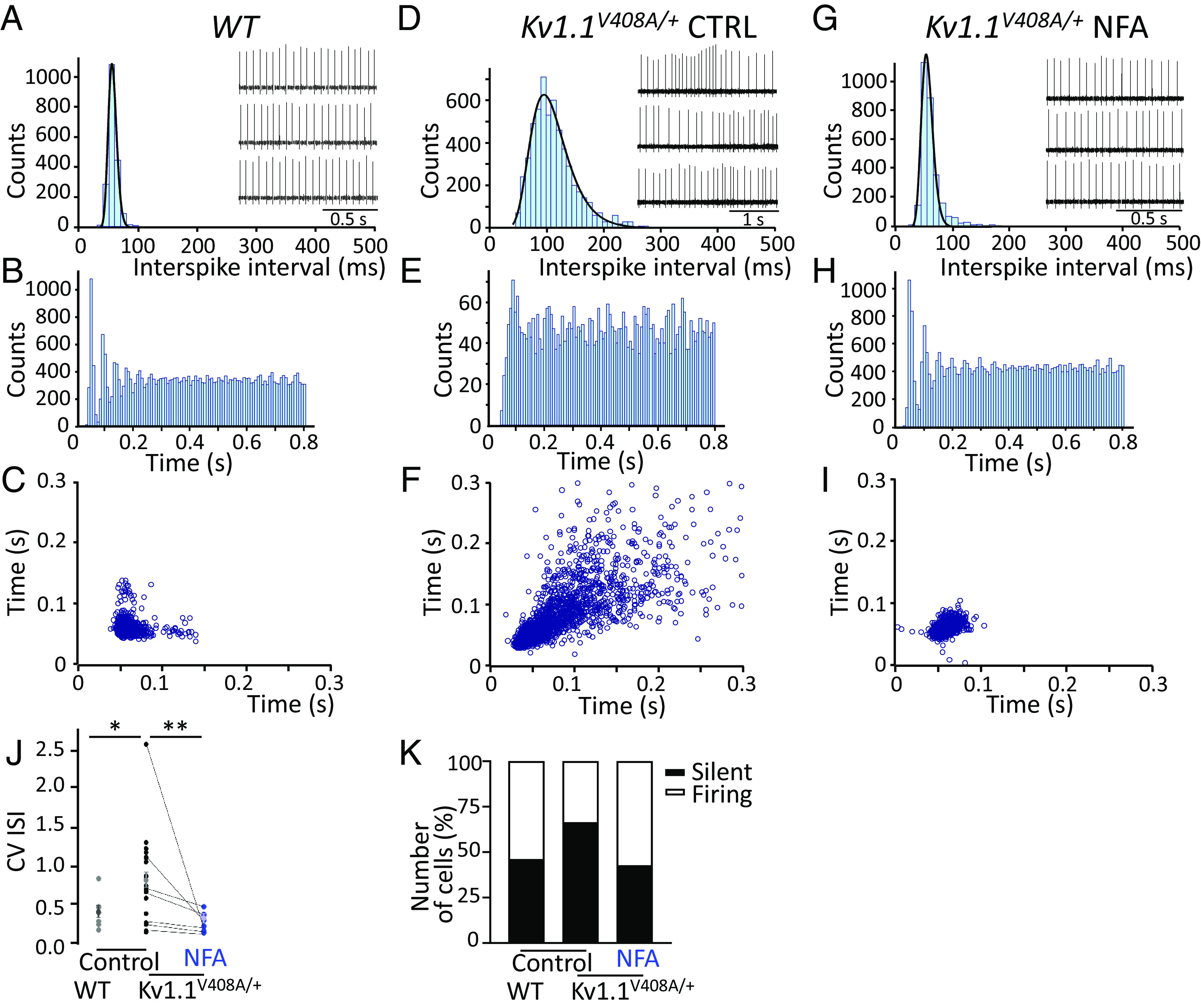
NFA ameliorates the firing properties of Purkinje cells recorded from Kv1.1*^V408A/+^* mice. Representative ISI distribution (*A*), spike recording (*Inset*), ISI autocorrelogram (*B*) and, scatter plot (*C*) constructed for a PC recorded extracellularly from a cerebellar brain slice of a WT mouse and, respectively, for a sample *Kv1.1^V408A/+^* PC recorded and analyzed before (Control, CTRL) (*D*–*F*), and after the superfusion of 100 µM NFA (*G*–*I*). Note that the ISI distributions were fitted with a probability density function of the exponentially modified Gaussian distribution with variance values of 8.7 for WT (*A*), 45 for *Kv1.1^V408A/+^*/CTRL (*D*), and 15 after NFA treatment (*G*). (*J*) CV ISI calculated for WT and *Kv1.1^V408A/+^* PCs (*P < 0.01; unpaired *t* test). The latter PCs were recorded before (*Control*) and after NFA (100 µM) application (mean ± SEM; **P < 0.001; unpaired *t* test). Note that *Kv1.1^V408A/+^* PCs displayed an irregular firing compared to WT, which was ameliorated by NFA treatment. (*K*) Percentage of silent (*black bar*) or spontaneously firing (*white bar*) PCs recorded from WT and *Kv1.1^V408A/+^* mice before (*Control*) and, upon incubation with NFA (100 µM). The NFA-induced increase in the percentage of spontaneously fining PCs was statistically significant: *P < 0.01, **P < 0.001 unpaired *t* test.

The precision of the spike timing of the PCs is crucial for properly controlling motor coordination. We predicted that the BC-dependent increase in GABA*ergic* tone, which is also characterized by an abnormal temporal pattern of inputs impinging onto the PCs of *Kv1.1^V408A/+^* mice (10, 12, 35), decreases the precision of firing, and silences a significantly larger number of PCs. Notably, BC terminals make synaptic contact and release GABA onto axon hillocks of PCs (Fig. 3*A*), where action potentials are generated at lower thresholds. To investigate these possibilities, extracellular somatic spike recordings were performed from spontaneously firing PCs in acute cerebellar slices dissected from WT or *Kv1.1^V408A/+^* mice, while minimizing interference from the intrinsic excitability of PCs. Notably, PCs exhibit tonic pacemaker activity that is crucial for correctly encoding cortical cerebellar information to the deep cerebellar nuclei and other motor coordination areas (40). PCs also display burst firing alternating with periods of quiescence, although it has been shown that PCs may fire regularly in slice preparations in vitro at room temperature (RT) (41). To characterize the temporal structure of firing, we calculated the interspike interval (ISI) distribution, ISI autocorrelograms, scatter plots, and coefficients of variation (CV). The analysis of tonic firing of WT PCs, recorded at RT under control conditions, highlighted a pacemaker-like activity characterized by a bell-shaped ISI distribution (Fig. 4*A*), ISI autocorrelogram with discrete peaks (Fig. 4*B*), and clustered scatter plot (Fig. 4*C*). However, in most recordings performed on *Kv1.1^V408A/+^* PCs, we found that the firing pattern was significantly altered (Fig. 4 *D*–*F*). Changes in the precision of firing for *Kv1.1^V408A/+^* PCs can be appreciated by comparing the ISI distribution (characterized by a long tail toward higher values; Fig. 4*D*), lack of discrete peaks in the ISI auto–correlogram (Fig. 4*E*), highly scattered plot (Fig. 4*F*), and higher CV ISI values compared to WT (Fig. 4*J*). These findings suggest that the EA1 mutation, V408A, alters the firing precision of cerebellar PCs.

Next, we tested the hypothesis that NFA mitigates the abnormal firing pattern exhibited by PCs from *Kv1.1^V408A/+^* cerebellar slices, possibly by ameliorating the functional properties of Kv1.1–V408A channels (Fig. 2). Notably, bath application of NFA restored the pacemaker firing pattern of *Kv1.1^V408A/+^* PCs (Fig. 4 *G*–*J*). To rule out the possibility that NFA modulated the firing pattern of PCs by acting on ANO1 channels, MONNA (1 µM) was applied on cerebellar slices dissected from WT mice, while recording the action potentials from PCs. MONNA had no effect on the firing frequency and CV of PCs (*SI Appendix*, Fig. S12 *A* and *B*). These properties were also not affected by the co-application of MONNA (1 µM) and NFA (100 µM) (*SI Appendix*, Fig. S12 *A* and *B*), suggesting that Ca^2+^-activated chloride channels do not play a role in the NFA-mediated effect on PCs’ firing pattern.

Based on the notion that the V408A mutation enhances GABA release onto PCs (10, 12, 13), we hypothesized that stronger inhibitory inputs silence a larger proportion of PCs in the cerebellum of *Kv1.1^V408A/+^* compared to WT mice. Furthermore, NFA application should increase the number of spontaneously firing PCs by inhibiting GABA release. To investigate these possibilities, we used infrared differential interference contrast optics to position the tip of the electrode near the soma of visualized PCs and recorded action potentials extracellularly. We observed that the number of silent PCs was significantly increased in cerebellar slices of *Kv1.1^V408A/+^* compared to WT mice (Fig. 4*K*). Incubation of *Kv1.1^V408A/+^* cerebellar slices in artificial cerebrospinal fluid containing NFA reduced the number of silent PCs, increasing those firing spontaneously (Fig. 4*K*). Overall, these results indicate that Kv1.1 channel dysfunction caused by the V408A mutation alters the precision of firing and silences a significantly larger number of PCs in the *Kv1.1^V408A/+^* cerebellum. NFA treatment ameliorated the firing properties of PCs and restored the number of spontaneously active neurons to values similar to those of the WT.

### NFA Ameliorates the Motor Deficits of Kv1.1^V408A/+^ Mice, and the Motor Performance and Neuromuscular Transmission of *Sh^5^* Flies.

**Fig. 5.**
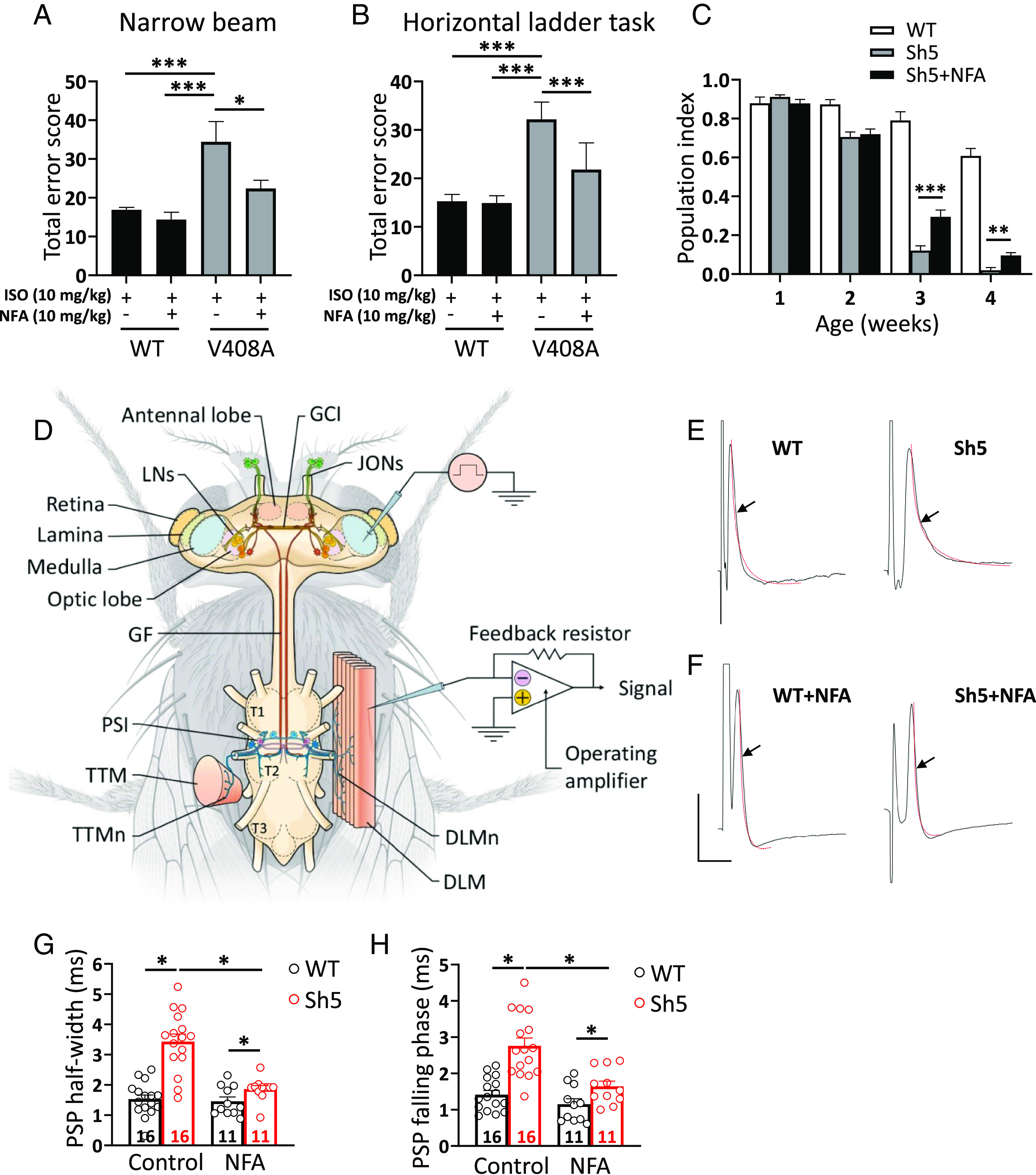
Administration of NFA ameliorates the motor performance and neuromuscular transmission in animal models of EA1. (*A*) Narrow beam test: motor balance and coordination were scored as the mice walked along a narrow beam (100-cm long, 0.75-cm diameter). (*B*) Horizontal ladder task: skilled walking was assessed by analyzing the ability of mice to cross a horizontal ladder with an irregular run pattern. To mimic stress-induced motor incoordination, mice were treated with ISO (10 mg/kg *i.p.*) and the behavioral tests were performed before and after the *i.p.* administration of 10 mg/kg NFA (n = 5; *P < 0.01; ***P < 0.001; one-way ANOVA). Note that NFA treatment ameliorated the motor balance, coordination and walking skill of *Kv1.1^V408A/+^ mice*. (*C*) Performance of *Sh^5^* flies in a climbing assay assessed at several time points throughout their adult life. Note that NFA significantly ameliorated locomotor dysfunction during the final weeks of the life of *Sh^5^* flies. Data are presented as the mean percentage climbing performance of flies±SEM of 10 independent experiments, and *n* ≥ 100 per condition. Significant differences between the two groups were tested by unpaired *t* tests (**P < 0.001, and ***P < 0.0001). (*D*) Schematic diagram showing the fly's thorax, the central nervous system, the GF system, the longitudinal muscle motor neurons (DLMn), and the DLM. To record PSPs the stimulating electrodes were placed in the eyes of the fly, while the recording electrode was placed in DLM (GCI: giant commissural interneuron; JON: Johnston organ; LN: lateral neuron; T1, T2, T3, pro-, meso-, and metathoracic neuromeres; PSI: peripherally synapsing interneurons; TTMn: tergotrochanteral muscle motor neuron; TTM: tergotrochanteral muscles; adapted from reference n. (42). (*E*–*H*) Effects of NFA treatment on evoked PSPs kinetics. Representative traces showing the typical time course of PSPs recorded from DLM of normal flies (WT) (*E*; *Left*) and *Sh^5^* flies (*E*; *Right*) upon stimulation of GF. The superimposed red curves show the fit of the falling phase with a single-exponential function from which the time constant was calculated (scale bar 10 mV/10 ms). Note the delayed falling phase of PSPs recorded from *Sh^5^* flies compared to WT. Effects of NFA treatment (NFA-supplemented diet for 17 to 20 d) on PSPs recorded from WT (*F*; *Left*) and *Sh^5^* (*F*; *Right*). (*G*) Bar graphs showing that the *Shaker* mutation prolongs the PSPs half-width of *Sh^5^* compared with WT flies calculated under control conditions. Note that NFA treatment corrected this effect. PSP half-widths were measured halfway (50%) between the baseline (just before the stimulus artifact) and the PSP peak. (*H*) Bar graphs showing that the *Shaker* mutation prolongs the time constant of the PSP falling phase of *Sh^5^* compared with WT flies calculated under control conditions. Note that NFA treatment corrected this parameter. The statistical analysis was performed using one-way ANOVA [falling phase duration, F (3,50) = 16.45, *P* < 0.0001; half-width, F (3,50) = 28.53, *P* < 0.0001] followed by Bonferroni’s post hoc test. Note the significant effect of the *Shaker* channel mutation F401I on both half-width and falling phase time constant of PSPs (*P < 0.0001; WT vs. *Sh*^5^); as well as the effect of NFA treatment that significantly decreased the half-width and sped up the decay time in *Sh^5^* flies (*P < 0.0001; *Sh^5^* vs. *Sh^5^*+NFA). The number of analyzed flies for every group is indicated inside each bar (data are mean ± SEM).

EA1 is characterized by stress-induced attacks of ataxia (9). *Kv1.1^V408A/+^* mice recapitulate this phenotype by showing a stress-induced loss of coordination (10). To investigate the potential therapeutic benefits of NFA on stress-induced motor dysfunction in *Kv1.1^V408A/+^* mice, skilled walking, limb placement, and motor coordination were assessed using well-validated behavioral tests, such as narrow beam and horizontal ladder tasks. Mimicking stress–fear responses by acute administration of isoproterenol (ISO) induced considerable adverse effects on the fine motor accuracy of *Kv1.1^V408A/+^* mice (Movie S1). In contrast, this treatment did not affect the motor performance of WT littermates (Fig. 5 *A* and *B* and Movie S2). Administration of NFA before injection with ISO (followed by motor exercise) ameliorated the motor performance of *Kv1.1^V408A/+^* mice, significantly reducing foot slips and missteps on both tests (Fig. 5 *A* and *B*).

Next, we assessed whether NFA alleviates the phenotypes of flies bearing a missense mutation in the *Shaker* Kv1.1 ortholog. We selected the *Sh^5^* mutant that bears the F401I amino acid substitution in the S5 region of the Kv1.1 channel (43, 44). Compared to WT flies, *Sh^5^* flies showed altered locomotor ability that resembles the EA1 phenotype (45). *Sh^5^* flies were raised on fly food supplemented with either NFA or the carrier as a control to investigate whether NFA ameliorated the locomotor defects of *Sh^5^* flies. NFA-treated flies showed a significant improvement in climbing ability during the final weeks of adulthood (Fig. 5*C*). Thus, NFA improved the climbing ability of *Sh^5^* flies from 12 to 30% at week 3 (*P* < 0.0001) and from 3 to 10% at week 4 (*P* = 0.0004) of their adult life (Fig. 5*C*), indicating that NFA ameliorates the *Shaker* mutant phenotypes in vivo.

Previous action potential recordings of *cervical giant axons* from *Shaker* (*Sh*) mutants and normal flies showed that the mutants exhibited abnormally long delays in K^+^ channel–dependent repolarization (46). This finding prompted us to investigate the neuromuscular junctions of *Sh^5^* mutant flies to assess potential mutation-induced changes in the function of *dorsal longitudinal muscle* (DLM) fibers. Postsynaptic potentials (PSPs) were evoked by electrical stimulation of the *cervical giant fiber* and recorded from the DLM fiber (Fig. 5*D*). The basic properties of PSPs, such as the duration, which is defined by its full width at half-maximum amplitude, and the time of the falling phase were analyzed in both mutant and normal *Drosophila* strains. Consistent with previous findings (46), evoked PSPs had an abnormal repolarization pattern in *Sh^5^* mutant flies, as shown by a significant increase in both PSPs duration and falling phase time compared to WT flies (Fig. 5 *E*, *G*, and *H*). Treating *Sh^5^* flies with NFA reversed both the abnormally increased width and falling phase time of PSPs to values similar to those of WT animals (Fig. 5 *F*, *G*, and *H*). In contrast, NFA treatment did not significantly change PSP parameters of WT flies (Fig. 5 *E*–*H*).

## Discussion

We have shown that NFA can enhance the activity of both Kv1.1 and Kv1.2 channels and offered a quantitative account for new mechanisms of action for NFA. The central finding of this study is that NFA can ameliorate EA1-induced dysfunctions in Kv1.1 channel gating, synaptic transmission, neuronal excitability, and motor deficits underlying the disease. Based on its effectiveness, NFA could be clinically exploited for pharmacological correction of defective Kv1.1 channels, which may represent a promising strategy to ameliorate multifunctional defects in individuals with EA1.

NFA increased Kv1.1-mediated currents by shifting the voltage dependence of channel activation toward more hyperpolarized potentials, an effect associated with an acceleration of ionic current activation, and slowdown of deactivation kinetics. Similarly, we found that NFA enhanced the activity of both Kv1.2 and Kv1.1/Kv1.2 channels, an interesting finding considering that Kv1.1 coassembles with Kv1.2 subunits in most brain structures (22). However, at depolarized potentials the effect of NFA on their current amplitudes was negligible. Thus, the evidence indicated that NFA does not increase the K^+^ current density for Kv1.1, Kv1.2, and Kv1.1/Kv1.2 channels after their open probability has reached its maximum. Gating charge measurements indicated that the effects of NFA on channel gating are likely caused by a drug-induced facilitation of voltage sensor activation, as demonstrated by the shift of the charge–voltage relationship toward hyperpolarized potentials and the marked slowing down of the OFF-gating current decay. While a coupling path functionally linking the S4 voltage-sensor movement to the outer selectivity filter, leading to slow (C-type) channel inactivation, has been proposed (47–49), in the case of Kv1.1–V408A, Peters and colleagues (50) have suggested that the mutant phenotype (i.e., flickering openings and faster inactivation) results from an unstable open state causing the activation gate to close at depolarized potentials (S6 helices). According to this view, the mechanical coupling between the voltage sensor (S4), open activation gate (S6), and pore is hindered and, the S6 slips in its closed conformation stopping current flow (50). We do not know whether NFA improves the interactions between the S4 and S6 helices. However, it appears to stabilize the open pore of Kv1.1–V408A mutant by facilitating voltage sensor activation; this action partially restores channel kinetics and slows down the inactivation process, counteracting the effects of the ataxia-causing mutation. High-resolution structures of Kv1.2-2.1 channels in the presence of NFA could shed light on the biophysical mechanism of action for NFA.

EA1 mutations have been consistently found to reduce the delayed-rectifier function by means of haploinsufficiency, shifting the voltage dependence to more depolarized potentials, accelerating deactivation and inactivation rates, and altering single-channel openings (9). While the V408A change does not affect the voltage dependence of the channel, the main deleterious defects exerted by this mutation are *i*) reduction in macroscopic current amplitudes with a minor change in channel expression at the plasma membrane (51); *ii*) acceleration of the deactivation kinetics (35); *iii*) shortening of the open duration of the channel, which becomes “flickery” (13), and *iv*) acceleration of slow inactivation kinetics (8). NFA ameliorates these gating defects and counteracts the smaller current amplitudes by increasing the availability of channels by enhancing their voltage sensitivity. EA1-affected individuals are heterozygous at the Kv1.1 locus, possessing a normal and a mutant allele which may be expressed, equally. Therefore, heteromeric channels composed of WT and mutated subunits may be formed. It has been proposed that the severity of impairment of the delayed-rectifier function in EA1 patients depends on both the type of mutation and the number of mutant subunits comprising the tetrameric Kv1.1 channels (13, 35). Notably, NFA exerted similar ameliorative effects on channels made-up of either four Kv1.1–V408A subunits or Kv1.1WT/Kv1.1–V408A subunits, indicating that this mutation does not curtail the activity of NFA. Although NFA appears to counteract most of the functionally relevant and common denominators of most EA1 mutation–induced channel dysfunctions, future studies should comprehensively characterize the effects of NFA on homomeric and heteromeric channel types harboring the hitherto-identified EA1 mutations, coexpressed with auxiliary subunits (52), to assess the broad applicability of this drug for EA1.

Kv1.1-containing channels appear to play a major role in GABA release at cerebellar BC terminals. Indeed, acute blockade of Kv1.1 channels with αDTx-K increased action potential–evoked Ca^2+^ influx, which is consistent with spike width as a major determinant of Ca^2+^ channel activation and total Ca^2+^ influx. Furthermore, both genetic and pharmacological inhibition of Kv1.1 led to a marked increase in evoked and spontaneous GABA release onto PCs, while miniature synaptic currents were unchanged, consistent with the supralinear relationship between Ca^2+^ influx and exocytosis (10, 12, 53, 54). Blockage of αDTx-sensitive K_V_1.1 channels resulted in unchanged rise and decay time constants of IPSCs recorded from PCs, further indicating a presynaptic site of the toxin action (10, 12, 53–55). Although BK_Ca_ channels are usually abundantly expressed at presynaptic terminals, a detailed immunohistochemical study identified cerebellar BC synapses as the sole exception to this rule (56). Here, we showed that the drug reduced the amplitude of IPSCs evoked by parallel fiber stimulation in WT PCs, as well as the amplitude and frequency of sIPSCs recorded from *Kv1.1^V408A/+^* PCs. NFA improved the gating properties of Kv1.1WT, Kv1.1*–*V408A, Kv1.1WT/Kv1.1*–*V408A, and Kv1.1WT/Kv1.2WT channels similarly. This evidence implies that NFA reduces the amplitude of eIPSCs in both WT and *Kv1.1^V408A/+^* PC to a similar extent. eIPSCs represent a readout of the evoked GABA release; thus, NFA-induced Kv1.1 and Kv1.2 current enhancement at the BC terminals likely results in reduced GABA release. A potential postsynaptic inhibitory effect of NFA, accounting for the reduced IPSC amplitude caused by the drug, could be confuted by the evidence that NFA conversely increases GABA-induced currents through the GABA*ergic* receptors composed of α1βY2 subunits, which are expressed natively in PCs or heterologously in oocytes (57–59).

PCs of *Kv1.1^V408A/+^* mice have a reduced frequency of firing (FF) due to the greater frequency and average amplitude of BC-mediated IPSCs than the WT (12). This increased GABA*ergic* tone reduces the precision of the ISI and the number of spontaneously active PCs, suggesting these as new pathogenic mechanisms underlying EA1. By increasing the noise of the baseline firing rate, erratic intrinsic pace-making is likely to reduce the accuracy of a PC in encoding and transmitting the strength and timing of the synaptic inputs it receives. This loss of information processing is likely to adversely affect cerebellar function. Here, we propose that NFA, by potentiating Kv1.1 and Kv1.2 channel activity at BC terminals, curtails GABA*ergic* signaling and increases the number of actively firing PCs, and the precision of the ISI. These actions could preclude abnormal cerebellar functions and account for the amelioration of the ataxic phenotype observed in NFA-treated *Kv1.1^V408A/+^* mice.

Kv1.1 channel expression has also been documented in granule cells (GC) in the cerebellum, where they contribute to A-type K^+^ channel currents (I_A_), which play essential roles in regulating spike timing, repetitive firing, dendritic integration, and plasticity (60). Based on our findings, we can formulate the following hypotheses: *i*) The V408A mutation in Kv1.1 channels modifies I_A_ in GC, altering their firing pattern, the GC–PF–PC synaptic transmission, and cerebellar plasticity, *ii*) NFA counteracts these mutation-induced defects by enhancing the activity of Kv1.1 channels in GCs and inhibiting T-type Ca^2+^ channels at PF–PC spines. Indeed, voltage-gated T-type Ca^2+^ channels are strongly expressed in PC dendritic spines of PF synapses and are inhibited by NFA (61). The activation of T-type channels generally requires a combination of hyperpolarization (to recover from steady-state inactivation) and depolarization. Perhaps the most obvious source of hyperpolarization in the PC is synaptic inhibition during periods of increased activity of the presynaptic BC and SC. The increased GABA*ergic* tone conferred to PCs by BCs expressing the mutated Kv1.1 channels, and the resulting hyperpolarization of PCs may relive the steady-state inactivation of T-type Ca^2+^ channels to some extent, thereby increasing their availability. NFA may counteract this process by inhibiting T-type Ca^2+^ channels found in PF–PC spines. Because Ca^2+^ entry through T-type channels can control synaptic plasticity at PF–PC synapses (62), the complex voltage-dependent mechanisms controlling T-type channel recruitment would contribute to the definition of specific and complex synaptic plasticity rules that depend on pre- and postsynaptic activity patterns, both of which could be disrupted by EA1 mutations in Kv1.1 channels and ameliorated by NFA treatment. These additional mechanisms could contribute to the pathophysiology of EA1 and improved motor performance displayed by NFA-treated *Kv1.1^V408A/+^* mice. These open questions will be addressed in future research.

*Shaker* flies carrying the F401I mutation (*Sh^5^*) in their Kv1.1 ortholog are behaviorally characterized by vigorous leg-shaking occurring in bursts, with action potentials displaying incomplete repolarization and leading to multiple spikes. Voltage clamp experiments of *Sh^5^* muscle, myotubes, and neurons showed that the I/V relationships for K^+^ current activation and inactivation shifted to more depolarized potentials, indicating that F401I produces functional defects similar to those caused by several EA1 mutations (9, 52, 63–65). The finding that NFA normalizes PSP kinetics in the DLM, tempts us to speculate that administering NFA to *Sh^5^* flies ameliorates their motor performance by amending the action potential shape by normalizing the voltage dependence of F401I channels expressed in muscle, myotubes, and neurons.

NFA is a potent analgesic and anti-inflammatory drug, and its neurobiological efficacy in mammals has been demonstrated to be more potent than dexamethasone, a widely used corticosteroid (66–69). The usual daily oral dosage of NFA results in a steady-state plasma concentration of approximately 70 µM (70–73). Because fenamates pass the blood–brain barrier efficiently (74), NFA is estimated to reach micromolar concentrations in the brain. Here, we show that NFA at similar and lower concentrations, in vitro, exerts significant ameliorative actions on Kv1.1 and Kv1.2 channel properties and cerebellar circuitry. It has been shown in the frog myelinated axon that a 5-mV shift toward the hyperpolarizing direction in the activation of K^+^ currents is equivalent to increasing the number of available K^+^ channels by a factor of three (75). The NFA-induced negative shift of Kv1.1 activation V_1/2_ should be adequate to counteract the reduction in channel availability induced by most EA1 mutations. Indeed, all missense mutations identified to date are heterozygous and seldom result in a *dominant negative effect* (i.e., >50% reduction in current amplitude) (9, 76). Here, we neither estimated the concentration of NFA in the cerebellum of mice or cervical GF of flies treated with the drug, nor provided evidence showing the direct action of NFA on Kv1.1 containing channels expressed in the relevant neural circuits. Furthermore, we did not compare the pharmacodynamics of NFA administered to mice orally, intraperitoneally, or intravenously, which may be clinically relevant. Therefore, the assumption that NFA increases the availability of K^+^ channels in vivo to a level suitable for antagonizing the defects induced by V408A or F401I mutations is exclusively supported by the significant effect of NFA on the motor performance of *Kv1.1^V408A/+^* mice treated intraperitoneally or *Sh^5^* flies treated orally with the drug. In addition to enhancing the function of homomeric and heteromeric Kv1.1 and Kv1.2 channels, NFA may improve neuronal excitability, synaptic transmission, and motor performance via other pathways or by modulating the activity of other channel types, which do not undermine the potential clinical relevance of the drug. However, it should be pointed out that NFA affects the activity of several of these channels at millimolar concentrations (77–80), which is therapeutically irrelevant.

Overall, the evidence we provided here suggests that NFA may be a drug candidate to be clinically repurposed and tested in a clinical trial for patients with EA1. However, individuals with *KCNA2* disorders characterized by loss-of-function mutations in Kv1.2 channels (81–83) could also benefit from variant-tailored NFA therapy. Promptly identifying the potential side effects is critical. Specific studies carried out in several mammalian species, including non-human primates, showed the long-term tolerability of NFA and lack of negative effects on the central nervous and cardiovascular systems. Furthermore, experiments performed using left ventricular myocytes dissociated from pig and sheep hearts showed that high concentrations of NFA (300 µM) induced a very small current in only two out of four cells; hence, the effect of NFA on cardiac action potentials was not tested (84). Although this evidence collectively did not indicate any adverse effects of NFA on cardiac function, we performed whole-cell patch clamp experiments in a current-clamp configuration mode to record the action potentials of ventricular myocytes dissociated from murine hearts. These recordings carried out under control conditions and after drug application showed that NFA slightly prolonged the duration of action potentials of cardiac ventricular myocytes (*SI Appendix*, Fig. S13). These data disagree with previous findings; however, they could be accounted for the remarkable differences in excitation–contraction coupling of heart muscles from different species. Nevertheless, our data imply that any potential clinical trials using NFA must be performed under strict cardiovascular monitoring.

Generally, patients with EA1 experience a poor quality of life, as these individuals suffer from psychological disturbances resulting from the constant fear of facing unpredictable and possibly life-threatening attacks. Furthermore, some sufferers display invalidating atypical symptoms, including epilepsy, malignant hyperthermia, and skeletal deformities, and as a consequence, tend to confine themselves at home and avoid social life and professional activity. EA1 is an orphan disease, and NFA could prove to be an appealing restorative therapy to normalize Kv1.1 channel activity and prevent ataxia attacks. The excellent safety profile of NFA in adults and infants, as well as its cost effectiveness, suggests that this drug could be tested in clinical trials for possible motor and extramotor benefits in individuals with EA1 and serve as a valuable model for drug discovery.

## Materials and Methods

TEVC recordings from Xenopus *laevis* oocytes were performed 1 to 5 d after Kv1.1 and Kv1.2 RNA injection using a GeneClamp 500 amplifier (Axon Instruments) as previously described (35, 85). For gating current measurements, we expressed in oocytes the *Shaker* N-terminal Δ6-46 deletion mutant channel that lacks fast inactivation and carries the W434F mutation that abolishes ion conduction (GenBank accession #M17211). The oocytes were voltage-clamped using the *cut-open oocyte* technique (33, 34). HEK293 cells were cultured in Dulbecco’s modified Eagle’s medium (DMEM, Gibco) at 37 °C with 5% CO_2_. Human Kv1.1 and Kv1.2 cDNAs were subcloned into the mammalian expression vector pCDNA_3_ and transiently transfected with the pEGFP-N vector using Lipofectamine 2000 (Invitrogen), according to the manufacturer’s instructions. N2a cells (ATCC, Manassas, VA, USA) were cultured in DMEM and subsequently cultured in differentiation medium containing 2% FBS and 20 μM of retinoic acid in DMEM, for 4 d. Patch clamp recordings were performed in a whole-cell configuration, at RT (21 to 23 °C). Sagittal cerebellar slices (300 μm) were prepared from heterozygous *Kv1.1^V408A/+^* or WT C57BL/6J mice (10 to 14-wk-old). Whole-cell voltage clamp experiments were made from PC somas visualized using infrared DIC and an Axon 200B amplifier (Axon Instruments) or MultiClamp 700B (Axon Instruments). Skilled walking, limb placement, and coordination of WT and *Kv1.1^V408A/+^* mice were assessed using a horizontal ladder and narrow wooden beam, as previously described (86). The *D. melanogaster* strain used was the *Shaker* 5 (*Sh^5^*) mutant (43, 44). Flies were supplemented with food containing either the drug or vehicle. The climbing ability of the flies was measured weekly throughout adulthood. To record evoked PSPs, WT and *Sh5* flies were exposed to either vehicle- or NFA-supplemented food for 17 to 20 d and investigated as previously described (87–89). Evoked PSPs were recorded with an Axopatch 2-B amplifier (Axon Instruments).

## Supplementary Material

Appendix 01 (PDF)

Movie S1. The video shows the narrow beam test used to assess the motor coordination of a representative *Kv1.1^V408A/+^* mouse pretreated with ISO. The animal was placed at one extremity of the wooden narrow beam and allowed to walk on the beam. Note the frequent hindfoot missteps displayed by the mutant animal.

Movie S2. The video shows the narrow beam test performed using a representative WT mouse pretreated with ISO. The video highlights the typical motor coordination of a normal animal.

## Data Availability

All study data are included in the article and/or supporting information.
