# Supplementary material for: An activator of voltage-gated K+ channels Kv1.1 as a therapeutic candidate for episodic ataxia type 1
Source: Proc Natl Acad Sci U S A. Author manuscript; Available in PMC 2023 Aug 10. (PMC10401004; doi:10.1073/pnas.2207978120)
Supplement: Appendix 01 [file EMS184636-supplement-Appendix_01.pdf]

Supplementary Information for

An activator of voltage–gated K^+ channels Kv1.1 as a therapeutic candidate for episodic ataxia type 1

Ilenio Servettini[a], Giuseppe Talani[b], Alfredo Megaro[a], Maria Dolores Setzu[c], Francesca Biggio[d], Michelle Briffa[e], Luca Gugliemi[f], Nicoletta Savalli[g], Francesca Binda[h,i], Francis Delicata[j], Gilles Bru–Mercier[k], Neville Vassallo[e], Vittorio Maglione[l], Ruben J. Cauchi[e], Alba Di Pardo[l], Maria Collu[c,2,†], Paola Imbrici[m], Luigi Catacuzzeno[n], Maria Cristina D'Adamo[o], Riccardo Olcese[g,p] and Mauro Pessia[e,k,1]

[1]Corresponding authors: Maria Cristina D'ADAMO and Mauro PESSIA
Emails: dadamo@lum.it; mauro.pessia@um.edu.mt; mauro@uaeu.ac.ae

This PDF file includes:

Figures S1 to S13
Table S1
Legends for Movies S1 to S2

Other supplementary materials for this manuscript include the following:

Movies S1 to S2

Supplementary Information Text

Materials and Methods

Recordings of K^+ currents from *Xenopus laevis* oocytes

All channel subunits were subcloned into the oocyte expression vector pBF, which provides 5′– and 3′–untranslated regions from the Xenopus β–globin gene

flanking a polylinker containing multiple restriction sites. Capped mRNAs were synthesized in vitro by using the SP6 mMESSAGE mMACHINE kit (Ambion). The EA1 V408A mutation was introduced into the human Kv1.1 cDNA by using the QuikChange Site–Directed Mutagenesis Kit (Stratagene) according to the manufacturer's instructions. The nucleotide sequence of the mutant channel was determined by automated sequencing. Procedures involving *Xenopus laevis* and their care were in accordance with the regulations of the Italian Animal Welfare Act and were approved by the local Authority Veterinary Service; procedures were also in accordance with the National Institutes of Health *Guide for the Care and Use of Laboratory Animals* and approved by the Ministero della Salute (autorizzazione n. 08–2018UT). The minimal number of animals was used and an average of 1–2 animals per condition was utilized. Frogs underwent no more than two surgeries, separated by at least 3 wk as previously described (1). Briefly, frogs were anesthetized with an aerated solution of 3–aminobenzoic acid ethyl ester. V–VI *Xenopus laevis* oocytes were isolated, digested with collagenase, each injected with 50 nl cRNA (Nanoject, Drummond, Broomall, PA), and incubated at 16 °C in ND96 solution containing (mM): NaCl 96, KCl 2, $MgCl_2$ 1, $CaCl_2$ 1.8, HEPES 5, gentamicin 50 µg/ml. Cells were injected with human homomeric Kv1.1WT or Kv1.1–V408A, or Kv1.2WT cRNA (50 nl, 5 ng/µl) (1). All EA1 mutations, which have been identified so far, are heterozygous. In the heterozygous state both normal and mutant alleles may be expressed. Co-injecting equal amounts of Kv1.1WT and Kv1.1–V408A cRNA (1:1 ratio; 2.5 ng/µl each, 50 nl total injected volume) in oocytes allows for the co-expression of wild-type and mutant subunits thereby possibly mimicking the heterozygous state of the patient. The reagents were supplied by Sigma. Electrophysiological recordings and data analysis were performed as previously described (35). Briefly, *two–electrode voltage–clamp* (TEVC) recordings were performed on *Xenopus* oocytes at 21–23°C, 1–5 days after injection. A GeneClamp 500 amplifier (Axon Instruments) interfaced to a PC computer with an ITC–16 interface (Instrutech) was used. Microelectrodes were filled with 3 M KCl and had resistances of 0.1–0.5 MΩ. The recording solution contained (in mM) 96 NaCl, 2 KCl, 1 $MgCl_2$, 1.8 $CaCl_2$, and 5 HEPES, pH 7.4. Currents were evoked by voltage commands from a holding potential of -80 mV, as described in the figure legends. Briefly, to assess the effects of NFA on the voltage dependence of Kv1 channels, K^+ current families were evoked by 500 ms depolarizing steps from -80 mV to +60 , in 5-10 mV increments, from a holding potential of -80 mV. Subsequently, the membrane potential was repolarized to -50 mV to record tail currents. The peak amplitudes of tail currents were plotted as a function of the depolarizing pre–pulse potentials and fitted with a Boltzmann function from which the half–maximal activation voltage ($V_{1/2}$) and slope factor (*k)* were calculated. The normalized I_{tail}/V relationships were calculated before and after drug superfusion into the recording chamber. The recordings were filtered at 2 kHz and acquired at 5 kHz with Pulse software (HEKA Elektronik, Germany). Data analysis was performed using Origin (Microcal, v4.1) and PulseFit (HEKA Elektronik). Leak and capacitive currents were subtracted using a P/4 protocol. To determine the statistical significance, paired Student's test was used. p<0.05 was considered

significant. Patch–clamp recordings of oocytes were performed at room temperature using an Axopatch 200B amplifier (Axon Instruments). Oocytes were bathed in a solution containing 120 mM KCl, 1 mM $CaCl_2$, 11 mM EGTA, 10 mM HEPES (pH 7.3) and had resting membrane potentials (V_m) of 0 mV under these ionic conditions. Recording electrodes were pulled from borosilicate glass, dipped in sticky wax (Kerr, Emoryville, CA, USA) prior to polishing and had resistances of 3–8 MΩ. The pipette solution, used for single–channel recordings, contained 120 mM KCl, 10 mM HEPES, 200 µM $CaCl_2$ (pH 7.2). Patch–clamp recordings were performed in the cell–attached or inside–out configuration by stepping to various test potentials and assuming that the V_m of the cell was 0 mV. Junction potentials between bath and pipette solutions were properly nullified. Current traces at each holding potential were filtered at 1 kHz with a 4–pole low–pass Bessel filter and acquired at 5–10 kHz with a Pulse+PulseFit program (HEKA Elektronik GmbH, Germany). Channel activity was analyzed with Microcal Origin v4.1. To quantify the effect of NFA on Kv1.1–V408A mutant channels studied at the single channel level, we constructed open–channel current amplitude distributions from Kv1.1V408A single–channel recordings in the presence and absence of NFA, and interpreted these distributions in terms of fast transitions of the channel among an open state and an unstable closed state. Due to the filtering of the experimental recordings, these fast transitions would not be fully resolved, and because of their presence the current amplitude distribution will be distorted from a Gaussian to an asymmetric distribution approximated by the following beta function (36-38):

$$f(y) = y^{(a-1)}\,(1-y)^{b-1}/B(a,b)\ ,$$

Where a=α τ, b=β τ, and $B(a,b) = \int_0^1 f(y)dy$

y represents the current amplitude normalized to the current level observed under the same recording conditions for WT Kv1.1 channels (1.4 pA); α and β represent the $C \rightarrow O$ and the $O \rightarrow C$ transition constants for the fast, unresolved process. Finally, τ represent the time constant of the filter, that can be approximated by τ=0.228/f, where f is the 3–dB attenuation frequency of a Bessel filter (36). The function used to fit the single–channel amplitude distributions has been obtained by convolving beta functions resulting from a fast–flickering process with the current noise typical of our recording system, and fitting by eye the experimental data while changing the rate constants associated with the fast–flickering process.

For gating current measurements, we expressed in Xenopus oocytes the *Shaker* N–terminal Δ6–46 deletion mutant channel lacking fast inactivation and, carrying the W434F mutation that abolishes ion conduction (GeneBank accession #M17211). The oocytes were voltage–clamped using the *cut–open oocyte* technique (COVG) (33). The extracellular solution contained (in mM): 110 NMG–MES, 2 CaMES, 10 HEPES (pH=7.4); whereas the intracellular solution (in mM): 110 NMG–MES, 2 EDTA, 10 HEPES (pH=7.4). The oocyte was impaled with a glass pipette filled with solution containing (in mM): 3000 Na–MES, 10 NaCl, and 10 HEPES (pH=7). Leak and capacitive currents were subtracted using a P/-6

protocol. Note that higher concentrations of NFA were used in these experiments to demonstrate the relative small but significant effect of this drug on gating currents properties. Data for the gating charge (Q(V)) curves were fitted to the sum of two Boltzmann distributions where Q_{max} and Q_{min} are the maximum and minimum Q, respectively; z is the effective valence of the distribution; V_{half} is the half-activating potential; V_m is the membrane potential; F, R, and T are the usual thermodynamic constants. The time–course of ON and OFF gating current decay (for membrane potentials more positive than -40 mV) was fit to one exponential function as: f(t)= A•exp(-t/τ)+B, where A is the amplitude, t is time, τ is the time constant and B is the baseline.

Recordings of K^+ currents from HEK293 and N2a cells

HEK293 cells were cultured in Dulbecco modified Eagle medium (DMEM, Gibco) supplemented with 10% north American fetal bovine serum (Gibco), 2 mM L–Glutamine, 100 U/ml penicillin and 0.1 mg/ml streptomycin at 37 °C with 5% CO_2. Human Kv1.1 and Kv1.2 cDNAs were subcloned into mammalian expression vector $pCDNA_3$ and transfected singly or co–transfected at a 1:1 ratio using Lipofectamine 2000 (Invitrogen), according to manufacturer's instructions. Cells were also co–transfected with pEGFP–N vector. 24 and 48 hours after the transfection, macroscopic Kv1.1 or Kv1.2 or Kv1.1/Kv1.2 currents were recorded using the patch–clamp whole–cell configuration at room temperature. Pipettes were pulled from thin–walled borosilicate glass and had resistance of 3–5 MΩ. Currents and voltages were amplified using an EPC–10 amplifier (HEKA Electronics, Lambrecht, Germany) and analyzed with the Patch–Master and Origin 8 software. Petri dishes with transiently transfected HEK293 cells were superfused with extracellular solution containing (in mM): NaCl 150, KCl 4, $MgCl_2$ 1, $CaCl_2$ 2, HEPES 10 (pH 7,4 with NaOH). Pipettes were filled with an intracellular solution containing (in mM): KCl 144, EGTA 1, HEPES 10 (pH 7.2 with KOH). The final access resistances ranged from 5 to 10 MΩ. Membrane capacitance measurements were made by using the transient compensation protocol of Patch–Master.
N2a cells (ATCC, Manassas, VA, USA) were maintained in growth Dulbecco's Modified Eagle Medium (DMEM; Hyclone, Logan, UT, USA) containing 10% fetal bovine serum (FBS; Serum Source International, Charlotte, NC, USA) and 1% Penicillin–Streptomycin solution (Gibco, Rockville, MD, USA), in a humidified 5% CO_2 atmosphere at 37°C. When N2a cells reached 70–80% confluency, the medium was changed into a differentiation medium, containing 2% FBS and 20 μM of retinoic acid (RA) in DMEM, for 4 days. Differentiated N2a cells were maintained in a humidified atmosphere of 5% CO_2 at 37°C, and the differentiation medium was changed every 2 days. Neuronal differentiation was electrophysiological and morphologically evaluated through the presence of dendrites and axons. Patch–clamp recordings of macroscopic delayed–rectifier K^+ currents were performed in whole–cell configuration at room temperature (21–23°C). The extracellular solution contained (in mM): NaCl 125, KCl 4, $CaCl_2$ 2, $MgSO_4$ 1.2, glucose 10, TTX 0.3, $CdCl_2$ 0.2, HEPES 10 (pH 7.4 with NaOH). The

pipette solution contained (in mM): KCl 140, NaCl 4, $CaCl_2$ 0.02, EGTA 0.8, $MgCl_2$ 2, MgATP 4, HEPES 10 (pH 7.4 with KOH). Patch pipettes had a resistance of 4–6 MΩ. Stimulation, acquisition, data analysis, and curve fitting were performed with pCLAMP software (Axon Instruments, Burlin–game, CA) and ORIGIN (Microcal Software, Northampton, MA). A sampling interval of 10 µs/point and series resistance compensation of 60–70% were applied. Currents were filtered at 5 kHz.

Recordings of Purkinje neurons IPSCs evoked by parallel fibers stimulation and firing pattern

Cerebellar coronal slices were prepared from adult C57BL/6 mice (P28–P31). The minimal number of animals was used and an average of 6–8 animals per condition was utilized. Briefly, mice were anesthetized by exposure to isoflurane, decapitated and the cerebellum dissected in ice cold bubbled (95% O_2/5% CO_2) slicing medium containing (in mM): sucrose 246, KCl 4, $NaHCO_3$ 26, $CaCl_2$ 1, $MgCl_2$ 5, glucose 10 and kynurenic acid 1 mM. 300 µm thick slices were obtained with a vibratome (Microm 650 V) in ice cold slicing medium and transferred to a holding chamber at 35°C with bubbled artificial cerebrospinal fluid (ACSF) containing (in mM): NaCl 120, KCl 3, $NaHCO_3$ 26, NaH_2PO_4 1.25, $CaCl_2$ 2, $MgCl_2$ 1 and glucose 16. Patch–clamp recordings were performed at room temperature in a recording chamber continuously perfused with bubbled ACSF. Borosilicate glass pipettes were pulled by a vertical puller (Narishige PC–10) to a final resistance of 2–4 MΩ when filled with the following internal solution (in mM): Cs methanesulfonate 135, NaCl 6, $MgCl_2$ 1, HEPES 10, MgATP 4, Na_2GTP 0.4, EGTA 1.5, QX314Cl 5, pH 7.3. Purkinje cells (PCs) were clamped at 0 mV and parallel fibers–induced responses elicited by electrical stimulation delivered by a pipette positioned in the inner side of the molecular layer and distant (more than 300 µm) from the recorded neuron. The stimulation pipette was filled with the following solution (in mM): NaCl 120, KCl 3, HEPES 10, NaH_2PO_4 1.25, $CaCl_2$ 2, $MgCl_2$ 1, glucose 10, pH 7.3. A Grass Stimulator (S48) was used to deliver stimulating pulses with amplitudes ranging from 10 to 100 V. The duration of the pulse was 0.3 msec, and stimulation frequency was 0.5 Hz. Stimulation was applied every 20 seconds and 3–6 consecutive induced responses averaged before analysis. Series resistance was compensated by 80% and monitored through the experiment by applying a small hyperpolarizing step (-10 mV) before stimulation. Data were collected with the MultiClamp 700B (Axon Instruments), filtered at 2 kHz and digitized at 20 kHz.

Recordings of spontaneous IPSCs from cerebellar Purkinje neurons

Sagittal cerebellar slices (300 µm thick) were prepared from heterozygous *Kv1.1*$^{V408A/+}$ littermate C57BL/6J mice (10–14–week–old). Briefly, mice were first sedated by *i.p.* injection of chloral hydrate (4%, *i.p.*). After decapitation, the cerebellum was removed, and sagittal slices of the vermis were cut using a

Vibratome. Slices were incubated in ACSF at 35 °C for 30 min and then stored and recorded in ACSF at room temperature (21–23°C). Slices mounted in a chamber were perfused continuously with ACSF (2 ml/min) and bubbled with a 95% O_2/5% CO2 gas mixture. All experiments were performed within 4–5 h of completion of slicing to ensure viability. The composition of the ACSF solution was (in mM): 119 NaCl, 2.5 KCl, 1 NaH_2PO_4, 26.2 $NaHCO_3$, 1.3 $MgCl_2$, 2.5 $CaCl_2$ and 10 dextrose. PCs were recorded using an internal pipette solution consisting of (in mM): 140 CsCl, 1 EGTA, 10 HEPES, 1 $MgCl_2$ and 5 MgATP; pH 7.3 with CsOH. Whole–cell voltage clamp experiments were made from PC somas visualized with infrared DIC on a Zeiss Axioskope 2 upright microscope. PCs were distinguished from other cerebellar cells based on their distinct location, massive dendritic trees, large cell bodies (approximately 20 μm), characteristic pear shape attributable to the stump of the apical dendrite. Whole–cell patch–clamp recordings were made using an Axopatch 200B amplifier (Axon Instruments, Union City, California) interfaced to a PC with an ITC–18 computer interface (Instrutech Corp, Port Washington, New York). Electrodes pulled from borosilicate glass capillaries had resistances of 2–4 MΩ when filled with the internal solution. Seal resistances were 5–10 GΩ. The membrane was ruptured by further suction. The recordings were performed after ≥10 min of stable seal formation and were analyzed on condition that AP amplitudes were ≥80 mV and the series resistance changed less than 20% throughout the entire recording period. Complete exchange of the bath solution occurred in about 1–2 min. Data were collected at a sample frequency of 25 kHz and analyzed using Microcal Origin v4.1 software. Event detection was performed analyzing 30 sweeps of 2–s duration using Patch–master (HEKA Elektronik, Germany) and ORIGIN (Microcal Software, Northampton, MA) software. Bar graphs were made of two parameters extracted from the continuous records: the amplitude and the inter–event time interval. The amplitude of an event is defined as the difference between a short baseline segment just before the rapid current increase and the peak of the current. The inter–event interval is defined as the time between the onset of the current rise of consecutive events. The IPSCs control values were averaged and normalized to 1 and the fractional amplitude and frequency were calculated accordingly. The few recordings that did not give IPSCs were discarded. The experiments were performed "blinded" in which the genotype of the slice under study was known only by an independent observer and revealed only at the end of the event analysis. The APs were recorded using an Axopatch 200B amplifier (Axon Instruments). Spike detection and analysis of firing pattern was performed using a script developed by Dr. Massimo Pierucci using a Spike2 software (Cambridge Electronic Design, Cambridge, UK). Individual spikes were extracted by utilizing a user–defined waveform. The events were visually inspected and those that did not possess the correct waveform were excluded. The inter–spike intervals (ISIs) were calculated and three different plots were constructed using at least 1000 intervals within a representative firing period: *i*) ISI histograms; *ii*) ISI auto–correlograms using the inbuilt auto–correlogram function in Spike2 and, *iii*) ISI scatter plots in which each point represented 2 successive ISI values. A regular–firing cell was represented by a narrow symmetric ISI histogram with little

to no skewness, several well–defined peaks in the auto–correlogram, and a compact ISI scatter plot. An irregular–firing PC was represented by an asymmetric and skewed ISI histogram, a flat auto–correlogram (*or one with very few peaks*), and a dispersed ISI scatter plot. The coefficient of variation (CV) was evaluated as an additional measure of firing irregularity. CV is defined as the ratio of the standard deviation σ to the mean μ and was calculated accordingly: $CV=\sigma/\mu$.

The extracellular recordings of action potentials (APs) from PCs were performed from cerebellar sagittal slices at room temperature, using pipettes filled with ACSF and positioning the tip close to the soma of the recorded neuron. The extracellular recording pipette had a resistance of 3.2-3.5 MΩ when filled with ACSF. Following the recording of PCs baseline activity (5 minutes), MONNA 1 µM was bath applied for 10 minutes before adding NFA 100 µM (with MONNA still present). The impact of both drugs on PCs firing was evaluated over a period of 10 minutes and the quantification of the last 5 minutes of recoding under each condition (MONNA and MONNA+NFA) was included in the analysis. Only tonic firing cells were included in the analysis. Data and statistical analyses and graph plotting were performed with Clampfit and GraphPad Prism v9.1 (GraphPad software, CA, USA). A Kolmogorov–Smirnov test was carried out on each data set to test for a normal distribution of data. Statistical tests were carried out using representative 60 s periods of the raw data. Data were presented as the mean±SEM and unpaired t–test was subsequently performed to compare the datasets before and during drug application.

Assessment of motor coordination and balance in mKv1.1$^{V408A/+}$ mice

All *in vivo* experiments were carried out in 5–month–old *Kv1.1*$^{V408A/+}$ mice, and in age– and gender–matched wild–type (WT) controls. *Kv1.1*$^{V408A/+}$ mice genotyping was confirmed by PCR and performed at 4 weeks of age to determine study groups. Animals were housed in polycarbonate cages and maintained under temperature (22–24°C) and humidity–controlled (55%) conditions. Food and water were provided *ad libitum*. All the experiments reported in this study were performed on the same animal groups. The procedures that involved the use of animals were conformed to the guidelines for the Care and Use of Laboratory Animals published from Directive 2010/63/EU of the European Parliament and approved by the local Authority Veterinary Services and Ministero della Salute.

Drug administration: mice were weighed immediately before testing and administered by intraperitoneal (*i.p.*) injection of NFA and the β–adrenergic agonist, isoproterenol (ISO). Briefly, NFA was dissolved in 0.9% NaCl and administered at a dosage of 10 mg/kg fifty minutes before the testing. NFA administration preceded twenty–five minutes the injection of ISO (10 mg/kg) dissolved in 0.9% NaCl. Twenty–five minutes after ISO was administered, animals were tested for motor function assessment.

Fine–motor skills and coordination were performed using well–validated motor tests according to standard recommendations. All tests took place during the light

phase of the light–dark cycle. Five mice per experimental group were used in each test. All mice received training for 2 consecutive days before performing motor function measurements. Before training and testing, mice underwent a period of habituation to the testing room and equipment. Skilled walking, limb placement and coordination were all assessed by the horizontal ladder task as previously described (86). Briefly, mice were placed upon a horizontal ladder and the number of missteps and foot slips through the ladder was evaluated by using a well–established footfall scoring system (86). Motor coordination was tested on a 100–cm–long wooden narrow beam (0.75 cm wide) suspended 30 cm above the floor (86). In details, mice were placed at one extremity of the beam and allowed to traverse the beam from one extremity to the other for three times. The number of the hindfoot missteps was counted while mouse walked along the length of the beam. All the analyses were made by inspection of the video recordings frame–by–frame.

Drosophila *melanogaster* stocks and culture

Flies were cultured on a sugar agar base and yeast paste (dry bakery yeast dissolved in milliQ water) in 50ml plastic vials at an incubation temperature of 25°C under 12:12 hours light–dark cycle. The strain utilized was the *Shaker* 5 (*Sh*^{5}) mutant, which carries a missense mutation in the S5 region of the *Shaker* locus resulting in the F401I amino acid substitution (42,43). NFA was dissolved in 91.4% ethanol and then diluted into yeast paste to result in a final concentration of 0.06% drug added per 1g yeast. The concentration was selected after performing preliminary dose–finding studies to identify an effective but non–toxic concentration.

Assessment of climbing activity

Fly climbing ability was measured every week throughout adulthood. In brief, flies were transferred to a clean vertical column (25 cm long, 1.5 cm diameter) with a conical bottom end. After tapping, flies reaching the top and remaining at the bottom of the column after a 45 second period were counted separately. Vertical climbing entails a strenuous activity because flies have to work against gravity to climb to the top of the column. Four trials were performed at 1–minute interval for each experiment. Only the last 3 trials were taken into consideration. Scores recorded were the mean number of flies at the top (n_{top}), the mean number of flies at the bottom (n_{bottom}) and the total number of flies assessed (n_{total}). The performance index (PI) defined as ½(n_{total}+n_{top}-n_{bottom}/n_{total}) was calculated and utilized to assess climbing performance.

Recordings of evoked post–synaptic potentials in flies

Flies from different experimental groups were anesthetized by using CO_2 and tightly anchored to a wax support with ventral side down, as previously reported (87-89) and visualized under a stereomicroscope. In order to activate the

Cervical Giant Fiber (CGF) of the fly, two tungsten stimulating electrodes connected to a stimulator (Master 8, A.M.P.I, Jerusalem, IL, USA) and triggered by a stimulus isolation unit (DS2A, Digitimer Ltd., Hertfordshire UK) – were placed into both eyes of the fly (Fig. 5*D*). Stimulus intensity was increased until the postsynaptic potential response was observed. Maximal stimulation intensity was not greater than 10 V. A third tungsten wire electrode was placed into the fly abdomen and used as ground signal. A borosilicate recording electrode–shaped by a horizontal puller (P97, Flaming Brown, Sutter Instruments, Novato, CA, USA) and with a resistance of 4–5 MΩ when filled with 3 M KCl was placed into the right or left backside of the fly along the 45a and 45b fibers of the Dorsal Longitudinal Muscle fibers (DLMs) (Fig. 5*D*). Evoked post–synaptic potentials (PSPs) were recorded with an Axopatch 2-B amplifier (Axon Instruments, Foster City, CA), filtered at 0.5 kHz and digitized at 1 kHz. PSPs were recorded in bridge mode, measured using peak and event detection software pCLAMP 9.2 (Axon Instruments, Foster City, CA), and analyzed off–line by Clampfit 9.2 software (Axon Instruments, Foster City, CA). All recordings were obtained from different flies belonging to each experimental group. Electrophysiological experiments were performed by applying a protocol consisting in a single CGF stimulation, delivered every 20 s, followed by PSP recording. PSPs falling phase time and half–widths (measured half–way between the baseline – just before the stimulus artefact – and the PSP peak) were measured in both WT and *Sh5* flies exposed to either vehicle or NFA–supplemented food for 17-20 days. Data are expressed as mean±SEM. and analyzed by one–way ANOVAs followed by Bonferroni's post–hoc tests.

Cardiac ventricular myocytes isolation and recording

Ventricular myocytes were enzymatically isolated from hearts of wild–type C57BL/6J male mice (ages 6–8 weeks). Briefly, mice were intraperitoneally injected with 5000 U/kg heparin (Millipore, Billerica MA, USA) and euthanized by cervical dislocation. The heart was quickly removed and mounted on a Langendorff apparatus followed by 3–min retrograde perfusion with 1.8 mM Ca^{2+} Tyrode's containing (in mmol/l): 140 NaCl, 5.4 KCl, 0.5 $MgCl_2$, 10 glucose, and 10 HEPES (pH 7.4) followed by 5 min perfusion with Ca^{2+}–free Tyrode's solution. Hearts were then perfused with the same Tyrode's solution but containing Liberase TH enzymes (0.025 mg/ml; Sigma–Aldrich) and bovine serum albumin (BSA; 1 mg/ml; Sigma–Aldrich). Ventricular tissue was then removed and minced, and ventricular myocytes were dispersed in KB solution containing (in mM): 80 KOH, 40 KCl, 25 KH_2PO_4, 3 $MgSO_4$, 50 glutamic acid, 20 taurine, 1 EGTA, 10 glucose, and 10 HEPES (pH 7.2 with KOH). Cells were stored at room temperature for at least 1 h before use. All chemicals used to make the solutions for cell isolations were obtained from Sigma–Aldrich.

Whole–cell patch–clamp recordings were performed from isolated ventricular myocytes at 21–23°C. Borosilicate glass pipettes (INTRACEL, Shepreth, UK) were used and microelectrodes had tip resistance of 2–4 MΩ when filled with

pipette's solution. The action potentials were recorded using an Axopatch 200B amplifier (Axon Instruments, Molecular Devices, San Jose, CA), sampled at 50 kHz and digitized with an Axon analog/digital converter (Digidata 1440A). The recordings were carried out in current current–clamp mode. The membrane potential was clamped at -80 mV and a current pulse of 40–60 nA lasting for 1 ms was sufficient to trigger an action potential, which was elicited at a frequency of 1 Hz. The bath solution contained (in mM): 126 NaCl, 5.4 KCl, 1.8 $CaCl_2$, 1.0 $MgCl_2$, 20 HEPES, and 11 glucose (pH = 7.4 with NaOH). The pipette solution contained (in mM): 90 Aspartic acid, 94 KOH, 26 KCl, 14 NaCl, 5.5. glucose, 1.0 $MgCl_2$, 10 EGTA, 5.0 MgATP, and 20 HEPES (pH = 7.2 with KOH). NFA was added to the bath solution and applied in the recording chamber by superfusion. Complete exchange of the solutions occurred in approximately 2 min. Data acquisition and analysis were performed using Clampfit software (version 10.7, Axon Instruments, Molecular Devices).

Statistical analysis

Data sets that passed the Shapiro–Wilk test for normality were analyzed with paired or unpaired Student's *t*–test. One–way ANOVAs followed by Bonferroni's post–hoc tests were used in distinct cases (additional details concerning the statistical analysis of data are reported in methods, figure legends and main text). Where appropriate the significance level of probability (*p*) for the difference between mean values are given.

Reference:

1. P. Imbrici, A. Cusimano, M. C. D'Adamo, A. De Curtis, M. Pessia, Functional characterization of an episodic ataxia type-1 mutation occurring in the S1 segment of hKv1.1 channels. *Pflugers Arch.* **446**, 373–379 (2003).

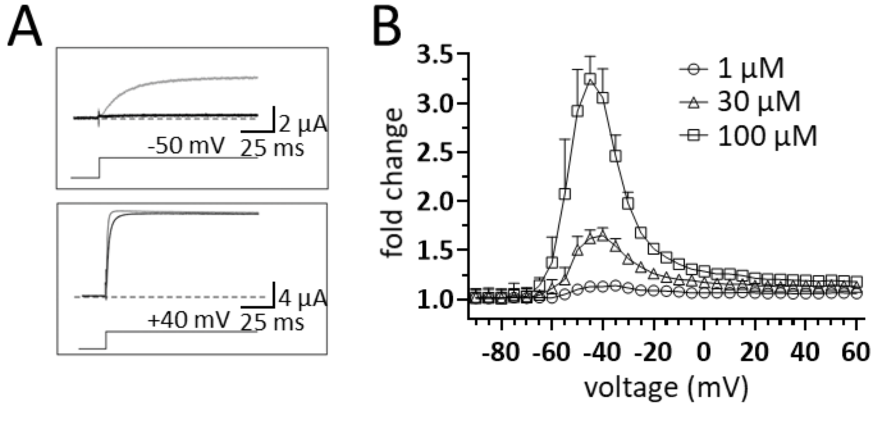

Fig. S1. **Niflumic acid increases Kv1.1 K^+ currents evoked at negative potentials.**
(*A*) Superimposed current traces obtained in the absence (*black*) and presence (*gray*) of 100 µM NFA and recorded at -50 mV (*upper traces*) and +40 mV (*lower traces*). (*B*) Plot of the mean fold increase of current as a function of step potential, calculated as the ratio of the current measured in the presence of NFA (at the indicated concentrations) to the current measured under control conditions. Note that NFA enhanced Kv1.1 currents several folds at negative potentials, while its effect at positive potentials was negligible (data are mean±SEM; n=6).

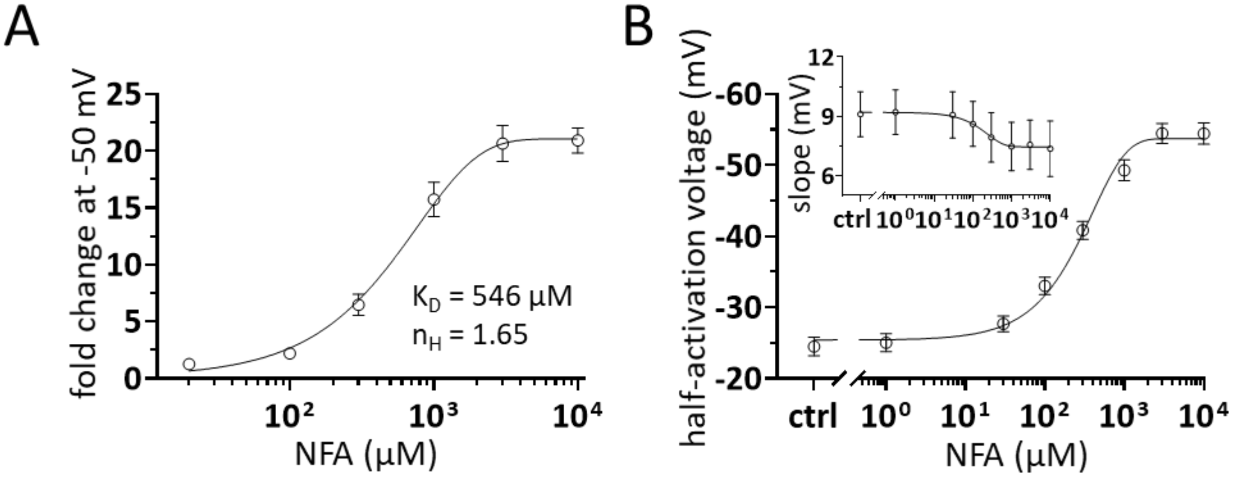

Fig. S2. **Niflumic acid dose–response relationships.**
(*A*) Plot of mean fold increase of Kv1.1 current recorded at -50 mV, as a function of NFA concentration. The solid line represents a fit of data points with a Hill equation, giving a dissociation constant (K_D) of 546 µM and Hill coefficient (n_H) of 1.65. Note that at this concentration the current was increased more than 10–fold. (*B*) Plot of half–activation voltage ($V_{1/2}$) as a function of NFA concentration. The solid line represents the best fit of data points with the equation $V_{1/2}$=V0+V1/(1+(K/[NFA])^n), with best fit parameters: V0=-24.9 mV, V1= -30.9 mV, K=272 µM, and n=1.05. The *inset* shows the plot of slope factor (mV), calculated using the Boltzmann equation, as a function of NFA concentration. Kv1.1 channels were expressed in *Xenopus* oocytes and K^+ currents were recorded using TEVC. Data are mean±SEM; n=7.

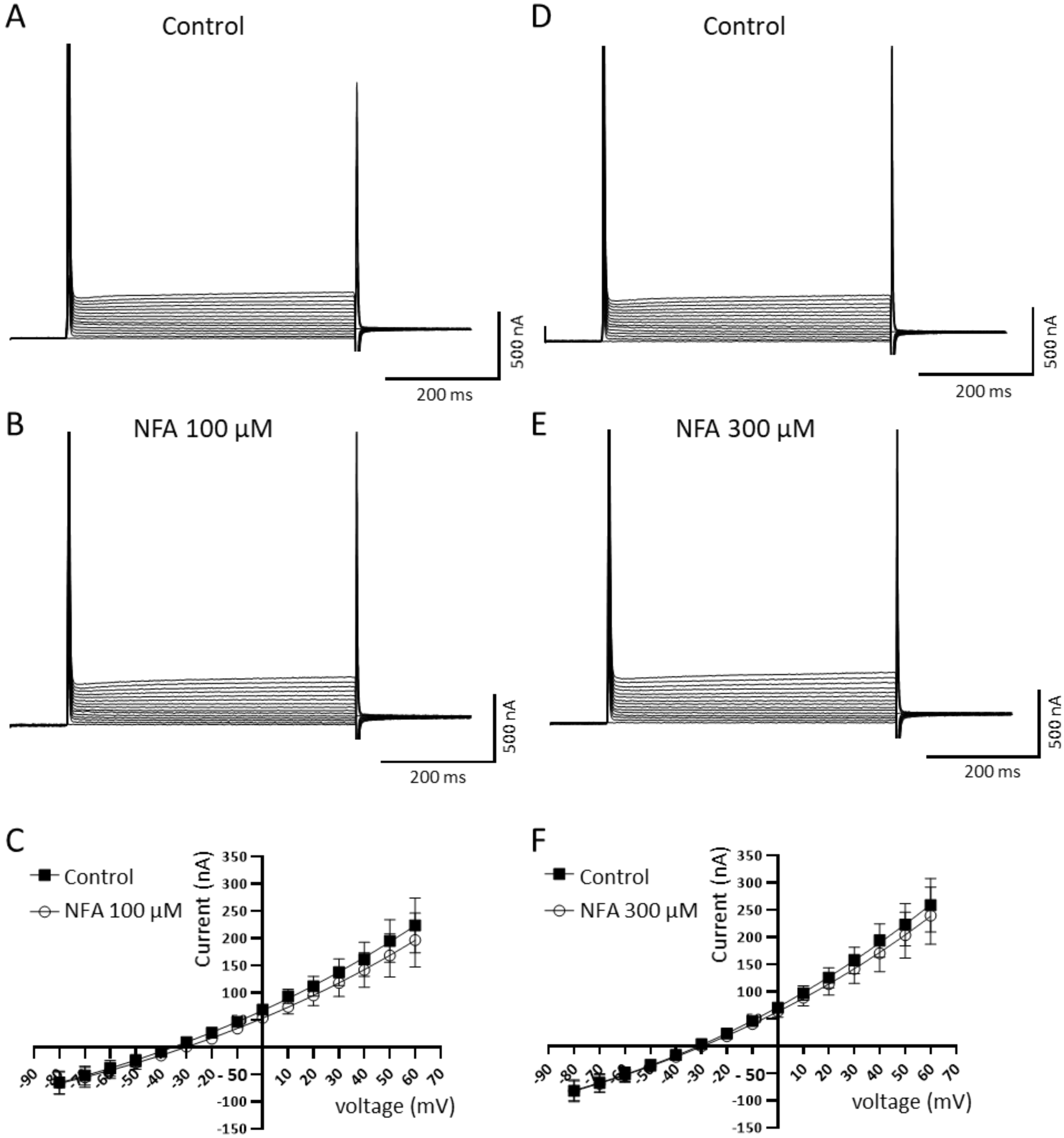

Fig. S3. **Niflumic acid does not affect the endogenous membrane currents expressed by *Xenopus* oocytes.**

Families of membrane currents recorded from oocytes injected with H_2O and performed in TEVC configuration under control conditions (*A*) and after the application of 100 µM NFA (*B*). Currents were elicited by voltage steps from -80 to +60 mV (ΔV= 10 mV; holding potential -80 mV). (*C*) Steady–state current as a function of voltage (IV relationships) plotted under control condition (*closed squares*), and after the application of 100 µM NFA (*open circles*). On the right–hand side, are reported sample families of current (*D,E*) and IV relationships (*F*) recorded and plotted under control condition after the application of 300 µM NFA.

Note that the drug exerts negligible effects on the endogenous currents. Data are mean±SEM; n=8.

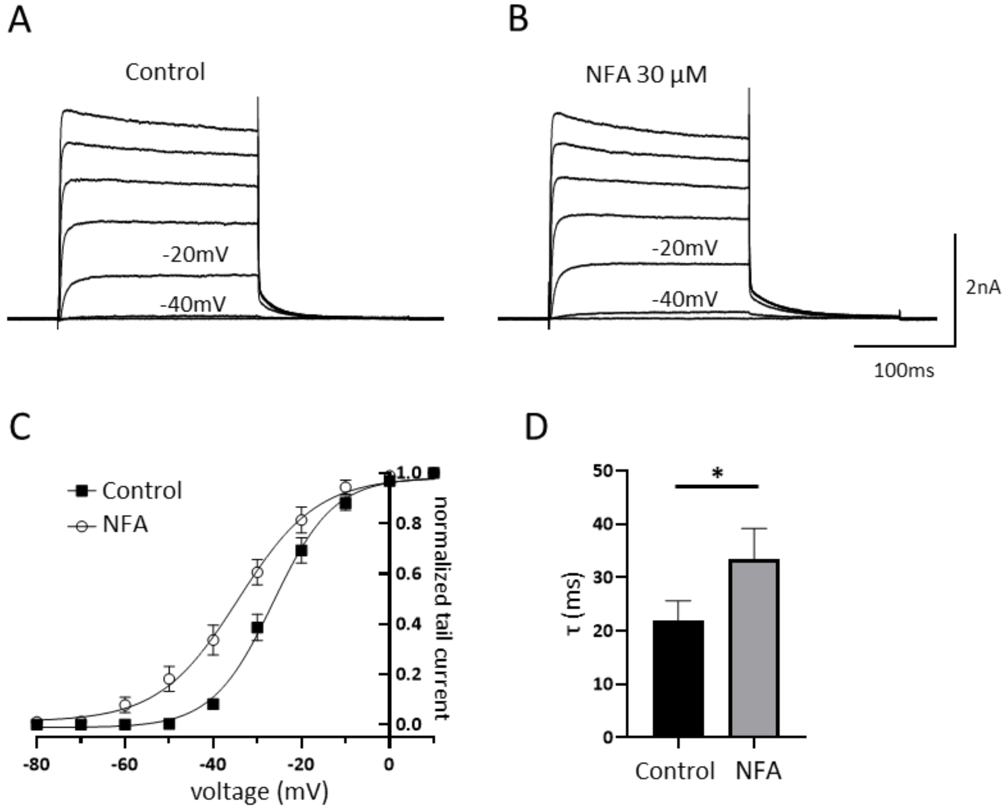

Fig. S4. **Niflumic acid enhances the voltage sensitivity of Kv1.1 channels expressed in HEK293 cells.**
(*A,B*) Representative families of current traces obtained by patch–clamp recording of HEK293 cells expressing Kv1.1 channels and applying depolarizing current steps from -80 mV to 60 mV (Δ=20 mV), from a holding potential of -60 mV, under control conditions (*A*) and after bath application of 30 µM NFA (*B*). (*C*) Normalized tail current amplitudes recorded at -60 mV from HEK293 cells expressing Kv1.1 channels and plotted as a function of depolarizing membrane potentials (ΔV=10 mV) under control conditions (*closed squares*), and after the application of 30 µM NFA (*open circles*). The solid lines represent fits of experimental data points with a Boltzmann relationship. The best fit parameters are: control (*squares*) $V_{1/2}$= -26.4 mV and k=6.5; NFA 30 µM (*circles*): $V_{1/2}$=-34.8 mV and k=9.1. (*D*) Bar graph showing the effects of 30 µM NFA on the deactivation time constants that were calculated by fitting a single–exponential function to Kv1.1 tail currents recorded at -50 mV. (Data are mean±SEM; n=6; *p<0.05, unpaired t–tests).

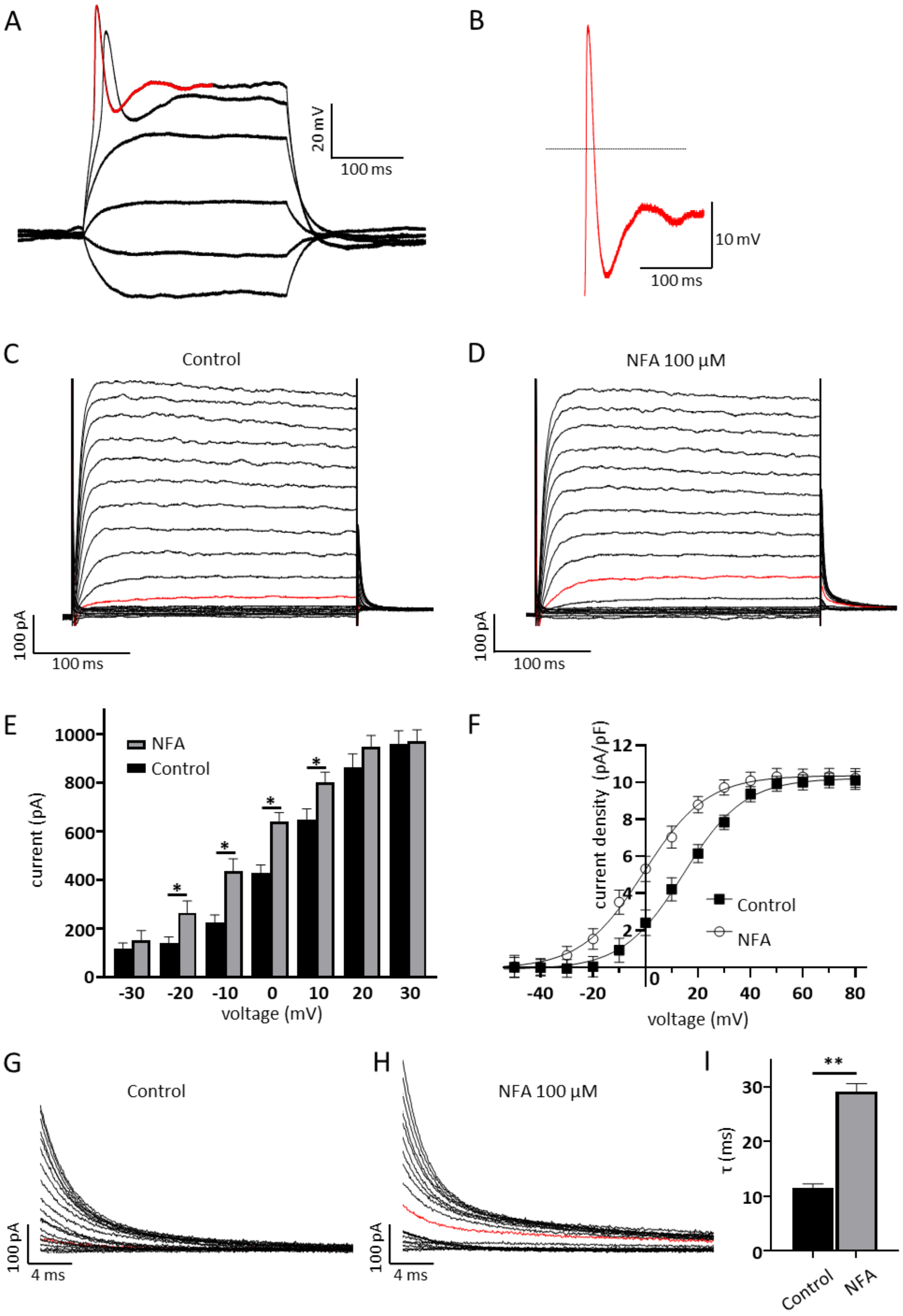

Fig. S5. **Niflumic acid enhances the voltage sensitivity of K+ currents natively expressed by N2a neurons.**
(*A*,*B*) Sample traces showing the recording of action potentials from neurons differentiated from N2a cells and carried out in current–clamp mode. (*A*)

Membrane potential changes elicited by applying -0.7- 2.3 nA, 300 ms pulses (the resting membrane potential was held at -70 mV by injecting -0.7 nA). (*B*) Enlargement of the action potential highlighted in A as a red colored trace (the dashed line indicates 0 mV level). Signals were sampled at 50 kHz and filtered at 5 kHz. (*C, D*) Representative families of K^+ current traces recorded from a cell in voltage–clamp mode by applying depolarizing current steps from -80 mV to 60 mV (Δ=20 mV) from a holding potential of -90 mV, under control conditions (*C*) and after bath application of 100 µM NFA (*D*). The red traces show the current recorded at -10 mV for direct comparison of the effect of NFA. Note that in addition to Kv1.1 channels, neurons differentiated from N2a cells also express Kv1.4 and Kv2.1 channels, endogenously (19). It is likely that homomeric Kv1.1, Kv1.4 and Kv2.1 channels, and heteromeric Kv1.1/Kv1.4 channels are formed in these cells and underlie the macroscopic outward current that were recorded and depicted in this figure. (*E*) Bar graph of mean current as a function of membrane potential calculated from cells recorded in control conditions (*black bars*) and upon application of 100 µM NFA (*grey bars*). (*F*) Tail current density recorded at -40 mV and plotted as a function of depolarizing step potentials (ΔV=10 mV) under control conditions (*closed squares*) and after the superfusion of 100 µM NFA (*open circles*). The solid lines represent fits of experimental data points with a Boltzmann relationship. The best fit parameters are: control $V_{1/2}$= 14.8 mV and k=11.3; NFA 100 µM: $V_{1/2}$=-0.4 mV and k=11.7. (*G,H*) Representative families of tail current traces recorded during the repolarization steps at -40 mV that were preceded by depolarizing steps from -80 mV to 60 mV (Δ=20 mV; holding potential -90 mV) and acquired under control conditions (*G*) and after bath application of 100 µM NFA (*H*). (*I*) Bar graph showing the deactivation time constants (τ) calculated at -40 mV under control conditions (*black bar*) and in the presence of 100 µM NFA (*grey bar*). The currents that were selected to calculate τ_s are shown in red and were elicited by pre–step potentials at +20mV, recorded at -40 mV and, fitted with a single–exponential function (data are mean±SEM, n=6; ***p*<0.01, unpaired t–tests).

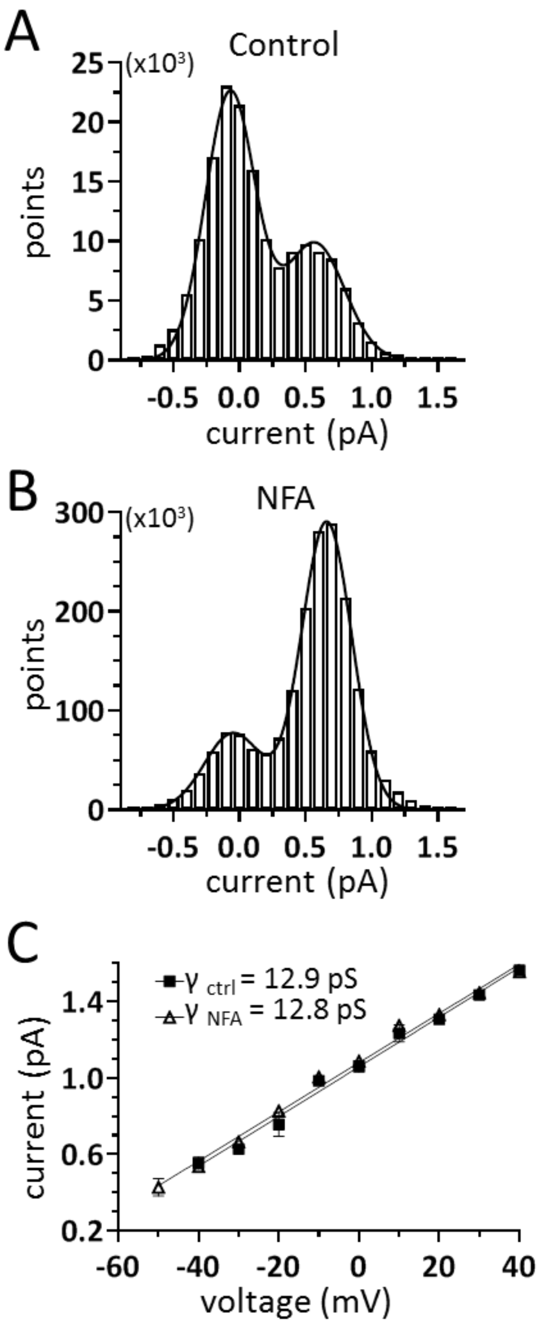

Fig. S6. Niflumic acid increases the open probability but not the Kv1.1 single-channel conductance

(*A*,*B*) *Point–by–point* current amplitude histograms constructed from Kv1.1 single channel recordings performed at -30 mV in the absence (*A, control*) and presence (*B*) of 100 µM NFA. The histograms were fitted with double Gaussian functions (*solid lines*) with the following best fit parameters; control: m_1=0; s_1=0.19 pA; A_1=1.07e^{-8} pA, m_2=0.7 pA; s_2=0.23 pA; A_2=5.75e^{-9} pA; NFA: m_1=0; s_1=0.22 pA; A_1=4.2e^{-8} pA, m_2=0.7 pA; s_2=0.19 pA; A_2=1.4e^{-7} pA. (*C*) Plot of mean single–channel current, as a function of applied voltages, assessed from single

channel recordings obtained in the cell attached configuration from oocytes expressing Kv1.1 in the absence (*closed squares*; n=5) and presence (*open tringles*; n=6) of 100 μM NFA in the pipette solution. The single–channel current was assessed from the double Gaussian fit of the amplitude histograms, as shown in panels *A* and *B*. The continuous lines in *C* represent the best fit of the experimental data points with straight lines, giving a slope/single channel conductance of 12.9 pS and 12.8 pS in the absence and presence of NFA, respectively.

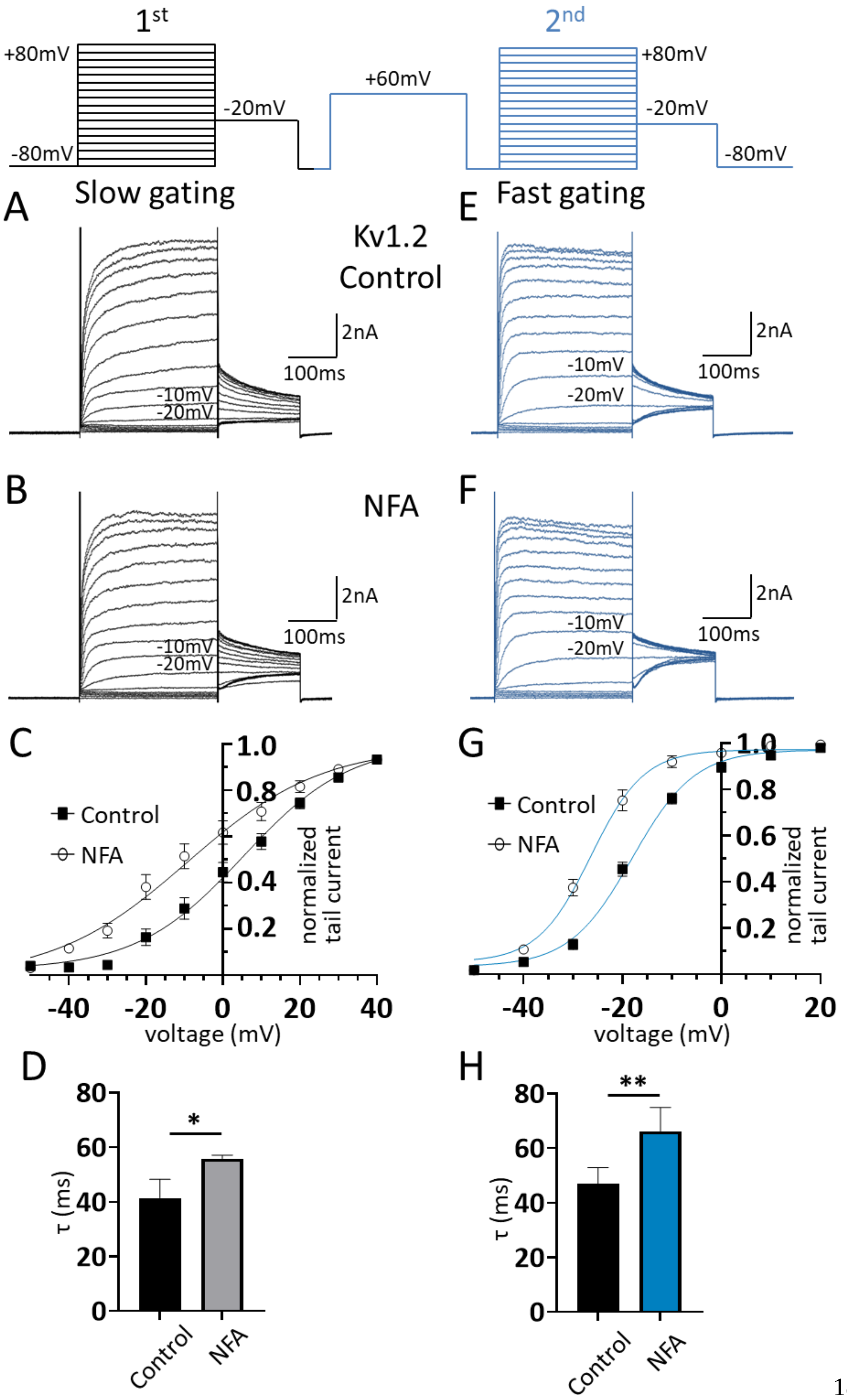
1st
+80mV
-20mV
-80mV
+60mV
2nd
+80mV
-20mV
-80mV
A
Slow gating
E
Fast gating
Kv1.2
Control
2nA
100ms
-10mV
-20mV
B
NFA
F
C
Control
NFA
normalized tail current
voltage (mV)
G
D
τ (ms)
Control
NFA
*
H
**

Fig. S7. Niflumic acid enhances the voltage sensitivity of the homomeric channels Kv1.2 exhibiting either the slow or fast–gating mode.
(*A*,*B*) Representative families of current traces recorded by using the whole–cell patch–clamp technique from HEK293 cells expressing Kv1.2 channels under control conditions (*A*) and after bath application of 100 µM NFA (*B*). Currents were elicited by applying depolarizing current steps from -80 mV to 80 mV (Δ=10 mV) from a holding potential of -80 mV, followed by a 150 ms voltage step at −20 mV to measure tail currents. This voltage protocol is shown above the current traces, as an inset colored in black, and labelled 1st. The Kv1.2 currents elicited by using these voltage steps exhibited a "*slow–gating mode*" (23,24). (*C*) Normalized Kv1.2 tail current amplitudes recorded at -20 mV, plotted as a function of depolarizing pre–pulse potentials (ΔV=10 mV), and recorded under control conditions (*closed squares*), and after the application of 100 µM NFA (*open circles*). The solid lines represent fits of the experimental data points with a Boltzmann relationship. The best fit parameters were: control (*squares*) $V_{1/2}$= 4.9±0.9 mV and k=14.2±0.8 mV; NFA 100 µM (*circles*): $V_{1/2}$=-8.8±1.4* mV and k=16.1±1.3 mV. (*D*) Bar graph showing the effect of 100 µM NFA on the deactivation time constants, which were calculated by fitting a single–exponential function to Kv1.2 tail currents recorded at -20 mV in the *slow–gating mode*. To favor the transition of Kv1.2 channels from the slow to fast–gating mode a combination of both the 1st (*black*) and 2nd (*blue*) voltage steps, interposed with a depolarizing pulse of 500 ms at +60 mV, was used (see inset reported above the sample traces) (23,24). (*E*,*F*) Representative families of current traces, exhibiting the "*fast–gating mode*", which were recorded from HEK293 cells expressing Kv1.2 channels under control conditions (*E*) and after bath application of 100 µM NFA (*F*). (*G*) Normalized Kv1.2 tail current amplitudes recorded at -20 mV, plotted as a function of depolarizing pre–pulse potentials (ΔV=10 mV), and recorded under control conditions (*closed squares*), and after the application of 100 µM NFA (*open circles*). The solid lines represent fits of the experimental data points with a Boltzmann relationship. The best fit parameters were: control (*squares*) $V_{1/2}$= -17.9 ±0.3 mV and k=6.2±0.3 mV; NFA 100 µM (*circles*): $V_{1/2}$=-26.2±0.4* mV and k=5.3±0.3 mV. (*H*) Bar graph showing the effects of 100 µM NFA on the deactivation time constants, which were calculated by fitting a single–exponential function to Kv1.2 tail currents recorded at -20 mV in the *fast–gating mode*. Data are mean±SEM; n=6; *p<0.05, ** p<0.01, unpaired t-tests.

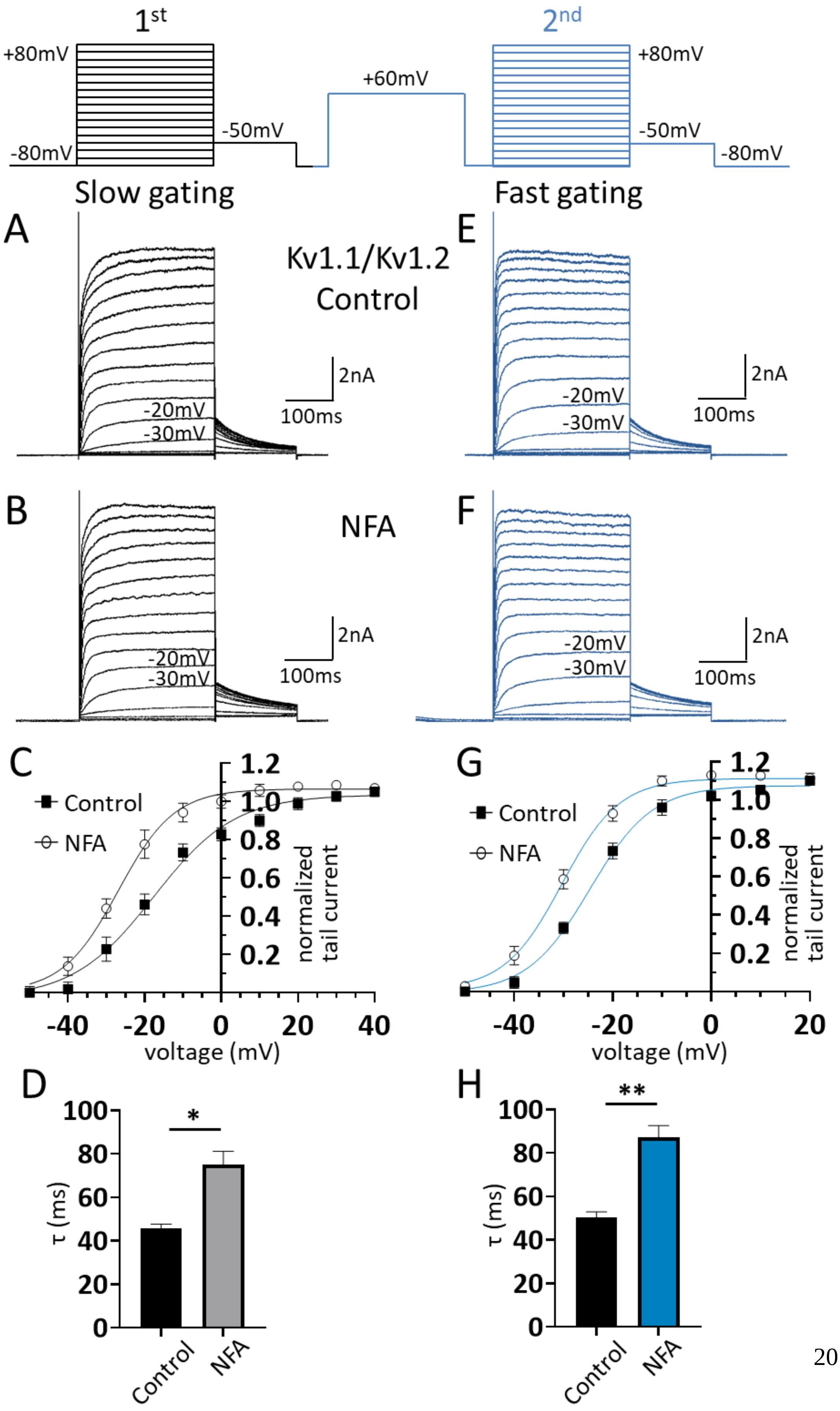
1st
2nd
+80mV
-80mV
-50mV
+60mV
+80mV
-50mV
-80mV
Slow gating
Fast gating
A
E
Kv1.1/Kv1.2
Control
2nA
100ms
-20mV
-30mV
B
F
NFA
C
G
Control
NFA
normalized
tail current
voltage (mV)
D
H
τ (ms)
Control
NFA
*
**

Fig. S8. Niflumic acid enhances the voltage sensitivity of the heteromeric channels Kv1.1/Kv1.2 exhibiting either the slow or fast–gating mode.
(*A*,*B*) Representative families of current traces recorded by using the whole–cell patch–clamp technique from HEK293 cells expressing Kv1.1/Kv1.2 channels under control conditions (*A*) and after bath application of 100 µM NFA (*B*). Currents were elicited by applying depolarizing current steps from -80 mV to 80 mV (Δ=10 mV) from a holding potential of -80 mV, followed by a 150 ms voltage step at −50 mV to measure tail currents. This voltage protocol is shown above the current traces, as an inset colored in black, and labelled 1st. The Kv1.1/Kv1.2 currents elicited by using these voltage steps exhibited a "*slow–gating mode*" (24). (*C*) Normalized Kv1.1/Kv1.2 tail current amplitudes recorded at -50 mV, plotted as a function of depolarizing pre–pulse potentials (ΔV=10 mV), and recorded under control conditions (*closed squares*), and after the application of 100 µM NFA (*open circles*). The solid lines represent fits of the experimental data points with a Boltzmann relationship. The best fit parameters were: control (*squares*) $V_{1/2}$= -16.7±1.1 mV and k=11.3±1.0 mV; NFA 100 µM (*circles*): $V_{1/2}$=-26.9±1.1* mV and k=8.2±0.9 mV. (*D*) Bar graph showing the effect of 100 µM NFA on the deactivation time constants, which were calculated by fitting a single–exponential function to Kv1.1/Kv1.2 tail currents recorded at -50 mV in the *slow–gating mode*. To favor the transition of Kv1.1/Kv1.2 channels from the slow to fast–gating mode a combination of both the 1st (*black*) and 2nd (*blue*) voltage protocols, interposed with a depolarizing pulse of 500 ms at +60 mV, was used (see inset reported above the sample traces). (*E*,*F*) Representative families of current traces, exhibiting the "*fast–gating mode*", which were recorded from HEK293 cells expressing Kv1.1/Kv1.2 channels under control conditions (*E*) and after bath application of 100 µM NFA (*F*). (*G*) Normalized Kv1.1/Kv1.2 tail current amplitudes recorded at -50 mV, plotted as a function of depolarizing pre–pulse potentials (ΔV=10 mV), and recorded under control conditions (*closed squares*), and after the application of 100 µM NFA (*open circles*). The solid lines represent fits of the experimental data points with a Boltzmann relationship. The best fit parameters were: control (*squares*) $V_{1/2}$= -25.3±0.5 mV and k=5.8±0.5 mV; NFA 100 µM (*circles*): $V_{1/2}$=-31.2±1.0* mV and k=5.1±0.9 mV. (*H*) Bar graph showing the effects of 100 µM NFA on the deactivation time constants, which were calculated by fitting a single–exponential function to Kv1.1/Kv1.2 tail currents recorded at -50 mV in the *fast–gating mode*. Data are mean±SEM; n=6; *p<0.05, **p<0.01, unpaired t-tests.

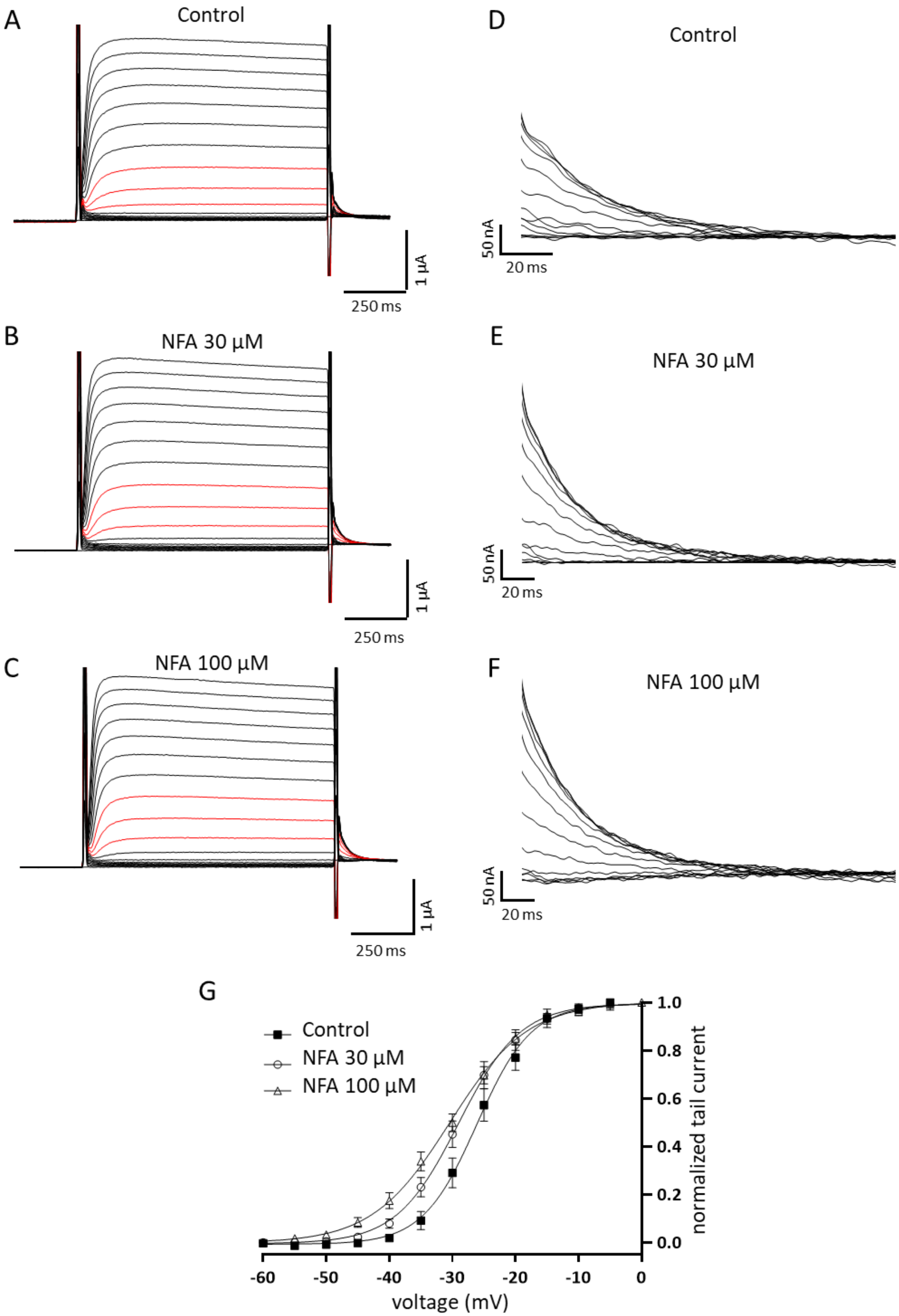

Fig. S9. **Niflumic acid enhances the voltage sensitivity of *Shaker* H4 mutant channels lacking the fast *ball–and–chain* inactivation.**

(*A*–*C*) Representative families of K^+ current traces recorded in TEVC mode from a *Xenopus* oocyte expressing the *Shaker* H4 channels under control conditions (*A*) and after the application of NFA 30 µM (*B*), or 100 µM (*C*). Currents were elicited by applying depolarizing steps from -80 mV to 20 mV (Δ=5 mV) from a holding potential of -80 mV. Tail current traces enlarged from the families of currents reported on the left–hand side and recorded under control conditions (*D*) and after the application of NFA 30 µM (*E*), or 100 µM (*F*). Tail currents were recorded during repolarization steps at -40 mV that were preceded by depolarizing current steps from -80 mV to 20 mV (Δ=5 mV; holding potential -80 mV). (*G*) Plot of normalized tail current amplitudes as a function of depolarizing step potentials and calculated under control conditions (*black squares*), and after the superfusion of NFA 30 µM (*open circles*), or 100 µM (*open triangles*). The solid lines represent fits of experimental data points with a Boltzmann relationship. The best fit parameters are control: $V_{1/2}$= -26.0 mV and k=4.2; NFA 30 µM: $V_{1/2}$=-29.0 mV and k=4.8; NFA 100 µM: $V_{1/2}$=-30.3 mV and k=6.1. Data are mean±SEM, n=6.

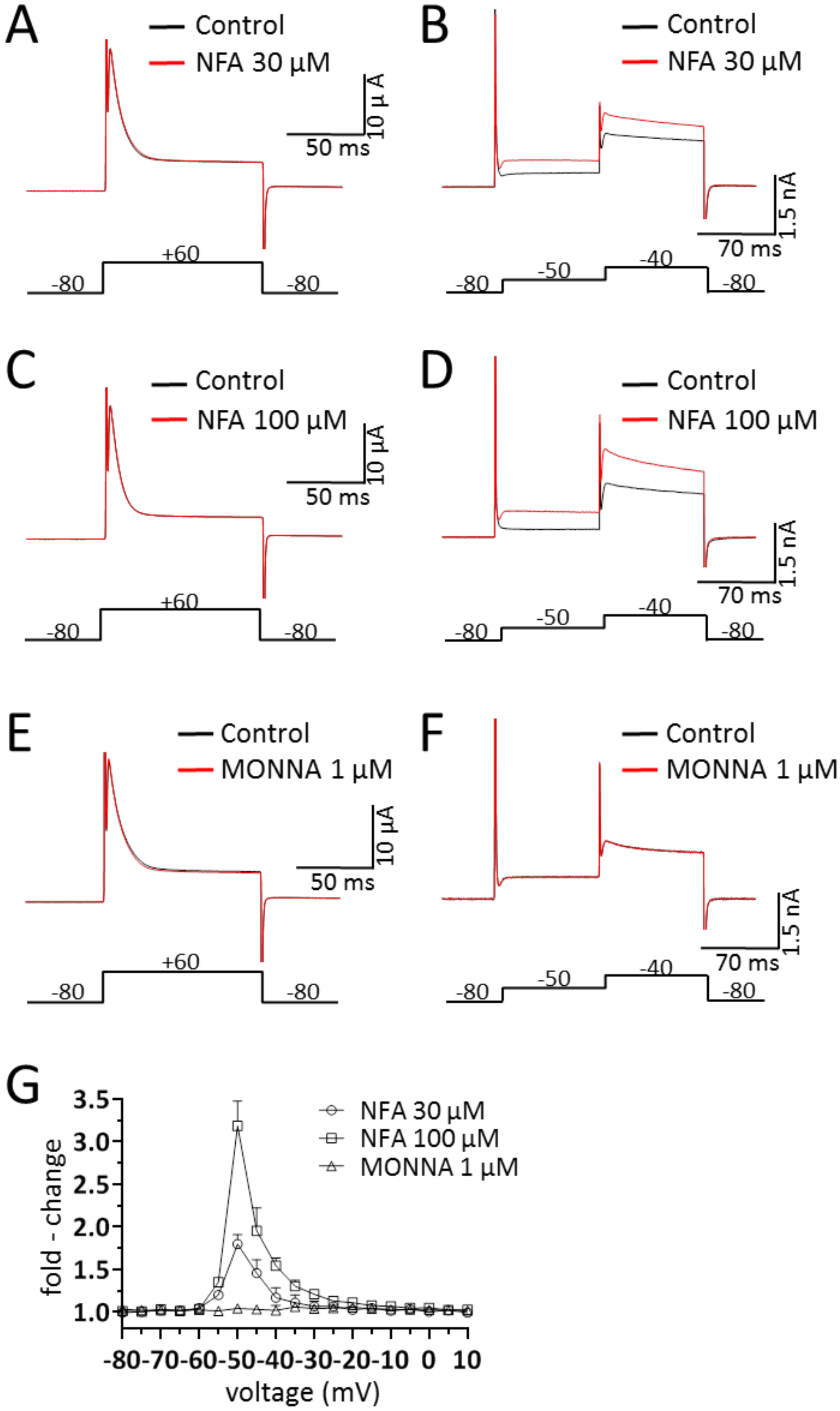
A
Control
NFA 30 µM
10 µA
50 ms
+60
-80
-80
B
Control
NFA 30 µM
1.5 nA
70 ms
-80
-50
-40
-80
C
Control
NFA 100 µM
10 µA
50 ms
+60
-80
-80
D
Control
NFA 100 µM
1.5 nA
70 ms
-80
-50
-40
-80
E
Control
MONNA 1 µM
10 µA
50 ms
+60
-80
-80
F
Control
MONNA 1 µM
1.5 nA
70 ms
-80
-50
-40
-80
G
NFA 30 µM
NFA 100 µM
MONNA 1 µM
fold - change
3.5
3.0
2.5
2.0
1.5
1.0
-80-70-60-50-40-30-20-10 0 10
voltage (mV)

Fig. S10. Niflumic acid increases the amplitude of WT *Shaker* K^+ currents at threshold potentials.

(*A,B*) Representative K^+ current traces recorded in TEVC mode from a *Xenopus* oocyte expressing WT *Shaker* channels under control conditions (*black trace*) and after the application of 30 µM NFA (*red trace*). Currents were elicited by steps from -80 mV to 60 mV (*A*) or from -80 mV to -50 and -40 mV (*B*), as shown in the inset reported below each sample trace (holding potential -80 mV). (*C, D*) Sample traces of WT *Shaker* currents recorded under control condition (*black trace*) and after the application of 100 µM NFA (*red trace*) (see A,B for experimental details). Note that the drug exerted negligible effects at depolarized potentials, while enhanced the WT *Shaker* current amplitude several fold at negative potentials. (*E, F*) Sample K^+ current traces recorded in TEVC mode from a *Xenopus* oocyte expressing WT *Shaker* channels under control conditions (*black trace*) and after the application of 1 µM MONNA (*red trace*). Currents were elicited as detailed above. Note that the drug exerted negligible effects on *Shaker* current amplitudes at all tested potentials. (*G*) Plot of mean fold of increase of WT *Shaker* currents, as a function of step potentials, caused by the application of NFA 30 µM (*circles*), NFA 100 µM (*squares*), or MONNA1 µM (*triangles*). The reported data represent the ratio of the current measured in the presence of the drug to the current measured under control conditions. NFA at both concentrations exerted a statistically significant effects at potentials between -55 mV and -40 mV (p<0.001; paired t–tests.). Data are mean±SEM; n=6–10.

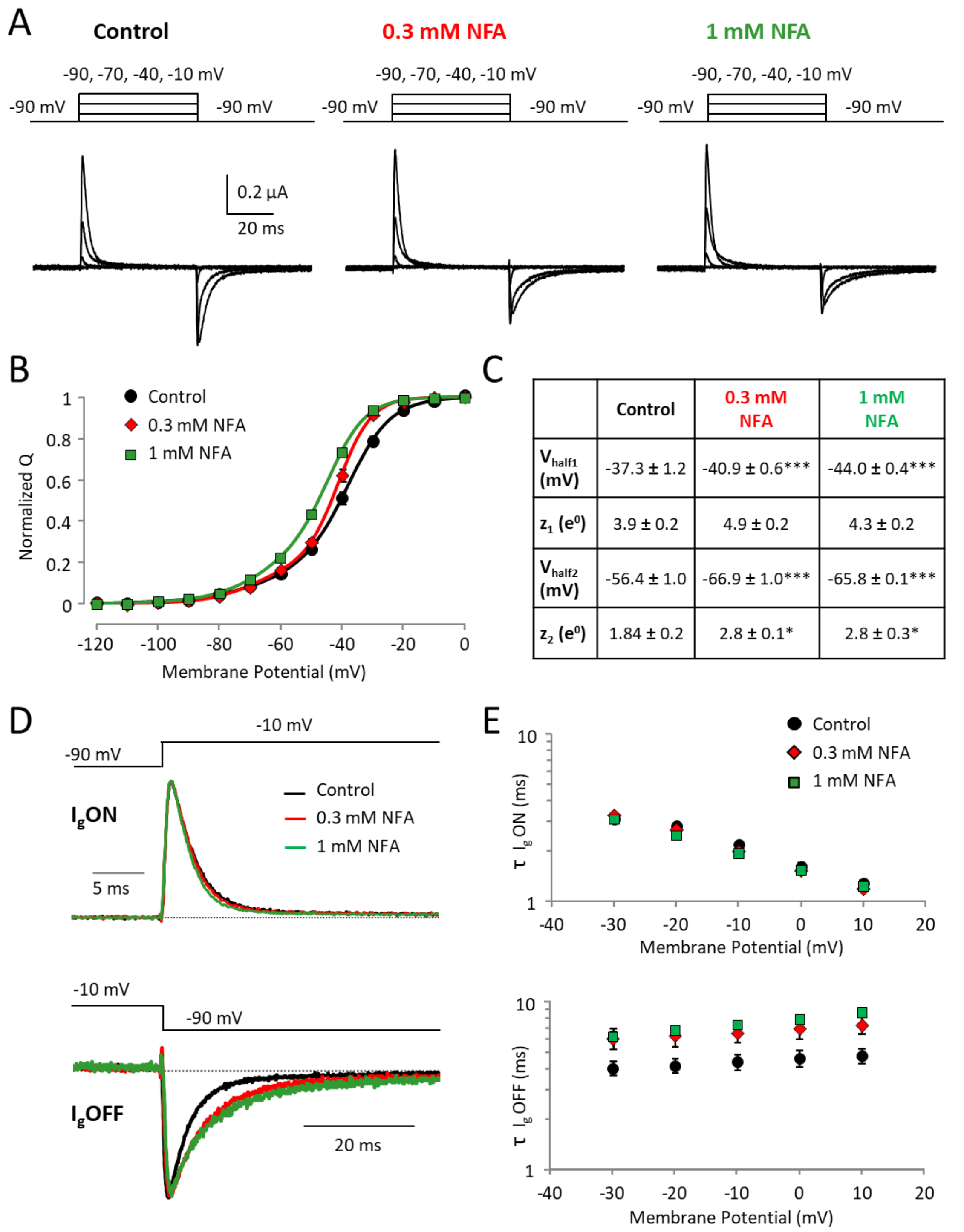

	Control	0.3 mM NFA	1 mM NFA
V_{half1} (mV)	-37.3 ± 1.2	-40.9 ± 0.6***	-44.0 ± 0.4***
z_1 (e^0)	3.9 ± 0.2	4.9 ± 0.2	4.3 ± 0.2
V_{half2} (mV)	-56.4 ± 1.0	-66.9 ± 1.0***	-65.8 ± 0.1***
z_2 (e^0)	1.84 ± 0.2	2.8 ± 0.1*	2.8 ± 0.3*

Fig. S11. **Niflumic acid slows the OFF–gating current decay and shifts the gating charge–voltage relationship towards more hyperpolarized voltages.** (*A*) Sample time courses of ON and OFF gating currents for *Sh–IR–WF* channels recorded at different membrane potentials (*the voltage steps are show on top*) under control conditions and after the superfusion of NFA, using the cut–open oocyte technique. (*B*) Plot of normalized gating charge Q (obtained by integrating

the $I_{g–ON}$) as a function of voltage and calculated under control conditions (*black circles*) and after the application of NFA 0.3 mM (*red diamond*), or 1 mM (*green squares*). The continuous lines represent the best fit of the experimental data with the sum of two Boltzmann distributions. (*C*) Table reporting the fitting parameters calculated under control conditions and after the application of NFA (mean±SEM; n=4–5). (*D*) Gating currents $I_{g–on}$ and $I_{g–off}$ recorded from *Sh–IR–WF* channels expressed in oocytes in response to a voltage step shown above the superimposed traces. Both $I_{g–ON}$ and $I_{g–OFF}$ were normalized to their peaks for directly compare gating current kinetics recorded under control condition (*black traces*) and in the presence of NFA 0.3 mM (*red trace*), or 1 mM (*greed trace*). (*E*) Plot of $I_{g–ON}$ (*upper panel*) and $I_{g–OFF}$ (*lower panel*) time constants, as a function of membrane potential, calculated under control conditions (*black circles*) and after the application of NFA 0.3 mM (*red diamond*), or 1 mM (*green squares*). Note that NFA shifts negatively the Q–V curve and slows down the $I_{g–OFF}$ decay. Data are mean±SEM, n=6; *P<0.05, ***P<0.001, paired t–tests.

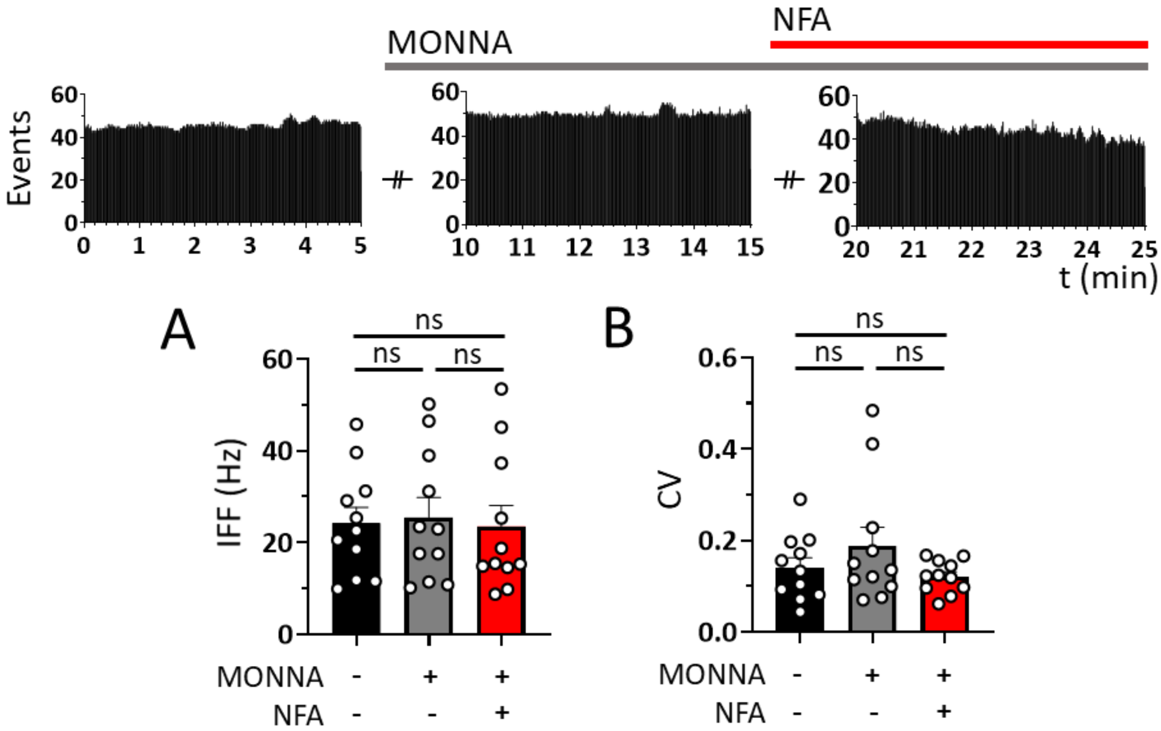

Fig. S12. MONNA and niflumic acid do not change the spontaneous firing activity of cerebellar PCs.
Representative rate histograms showing the spike discharge of a PC recorded extracellularly from a cerebellar slice dissected from a wild-type mouse in control conditions (*left rate histogram*), during the superfusion of MONNA (1µM; *middle rate histogram*), and during the co-application of MONNA (1µM) and NFA (100µM; *right rate histogram*). The grey and red horizontal bars show the time during which the drugs were superfused in the recording chamber. (*A*) Instantaneous firing frequency (IFF) and, (*B*) CV calculated in control conditions,

after the application of MONNA (1µM), and co-application of MONNA (1µM) and NFA (100µM). The slices were dissected from three mice, approximately 2–months–old (n= 11; n.s.: not statistically significant).

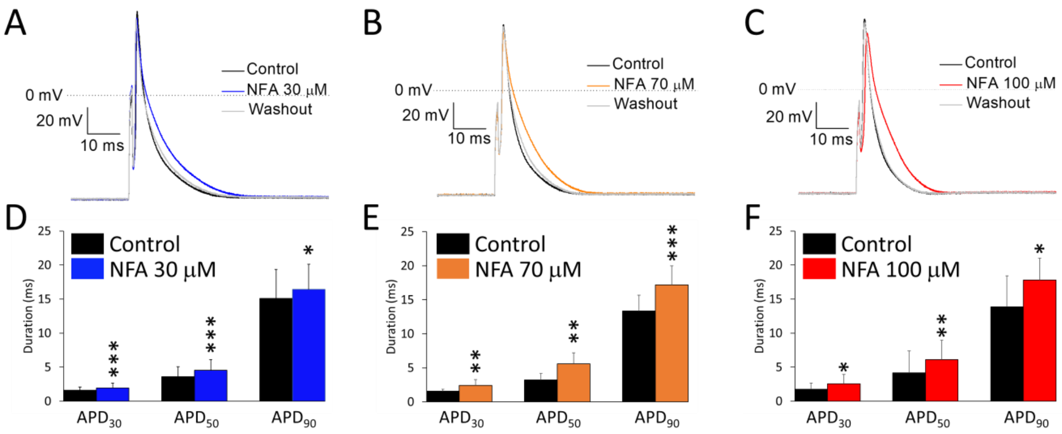

Fig. S13. Niflumic acid prolongs the duration of the action potential of murine ventricular myocytes.
Top panels: representative action potentials of a ventricular myocyte recorded by using the patch–clamp whole–cell configuration before (*control*), after the superfusion of NFA 30 µM (*A*), 70 µM (*B*), and 100 µM (*C*) in the recording chamber, and after drug washout. The dashed lines indicate 0 mV level. *Bottom panels*: bar graphs showing the mean action potential duration (APD) calculated at 30, 50, and 90% repolarization (APD_{30}, APD_{50}, and APD_{90}, respectively) before (*control*), after the superfusion of NFA 30 µM (*D,* n=19), 70 µM (*E, n=8*), and 100 µM (*F, n=7*) in the recording chamber. Data are mean±SD; **p*<0.05; ***p*<0.01; ****p*<0.001; paired t–test.

	Kv1.1-WT	Kv1.1-V408A	Kv1.1-WT+ Kv1.1-V408A	Kv1.2-WT
Voltage dependence				
$V_{1/2}$ (mV)	-24.6±0.2 (7)<br>-40.8±1.3 (7)***	-29.0±1.2 (6)<br>-41.9±0.8 (6)***	-24.9±1.0 (7)<br>-41.1±1.2 (7)***	-12.0±2.5 (4)<br>-24.6±2.5 (4)***
k (mV)	9.1±1.1 (7)<br>8.0±1.2 (7)***	9.4±0.2 (6)<br>6.6±0.2 (6)***	11.6±0.9 (7)<br>8.8±0.7 (7)***	9.1±0.6 (4)<br>7.0±0.7 (4)***
Activation kinetics				
τ_a at 0 mV (ms)	3.7±0.2 (4)<br>1.2±0.2 (4)***	3.20±0.30 (6)<br>1.33±0.06 (6)***	3.3±0.7 (7)<br>1.4±0.1 (7)***	5.2±0.4 (4)<br>1.8±0.1 (4)***
Deactivation kinetics				
τ_d at -60 mV (ms)	5.5±0.5 (4)<br>13.3±0.7 (4)***	0.40±0.02 (6)<br>0.86±0.11 (6)*	2.5±0.3 (5)<br>4.1±0.2 (5)***	4.5±0.1 (4)<br>5.8±0.2 (4)**
Slow inactivation				
τ at 0 mV (s)	τ_1=5.3±0.3<br>τ_2=27.1±7.8 (4)<br>τ_1=8.0±0.6 **<br>τ_2=48.4±3.4 (4)**	4.3±0.3 (10)<br>7.9±0.5 (10)***	7.0±0.3 (7)<br>9.8±0.4 (7)**	τ_1=1.99±0.11 (4)<br>τ_2=20.0±0.50 (4)<br>τ_1=2.03±0.16 (4)<br>τ_2=26.30±1.20 (4)**

Table S1. Voltage dependence and kinetics parameters for the indicated homomeric and heteromeric channel types calculated in control conditions and after the application of Niflumic acid. The voltage dependent and kinetics parameters of the indicated channels, expressed and recorded from *Xenopus* oocytes in TEVC mode, were calculated as described in the methods and captions of the figures. The data are tabled in black (*control*) and red (NFA 300 µM) colors. The number of the cells recorded is reported in brackets. The data are the mean±SEM; *p<0.05; **p<0.01; ***p<0.001; paired t–test.

Movie S1. The video shows the narrow beam test used to assess the motor coordination of a representative $Kv1.1^{V408A/+}$ mouse pretreated with ISO. The animal was placed at one extremity of the wooden narrow beam and allowed to walk on the beam. Note the frequent hindfoot missteps displayed by the mutant animal.

Movie S2. The video shows the narrow beam test performed using a representative WT mouse pretreated with ISO. The video highlights the typical motor coordination of a normal animal.
